# *Bacteroides thetaiotaomicron* (*BT6*) Restores Intestinal Homeostasis in *Escherichia coli* O157:H7-Challenged Mice

**DOI:** 10.3390/vetsci13040324

**Published:** 2026-03-27

**Authors:** Mohamed Osman Abdalrahem Essa, Nosiba S. Basher, Cheng Cheng, Saber Y. Adam, Nasir A. Ibrahim, Hosameldeen Mohamed Husien, Ahmed A. Saleh, Darong Cheng

**Affiliations:** 1 College of Veterinary Medicine, Yangzhou University, Yangzhou 225009, China; dh23054@stu.yzu.edu.cn (M.O.A.E.); 13291399018@163.com (C.C.); 2 College of Veterinary Medicine, Albutana University, Rufaa 22217, Sudan; 008643@yzu.edu.cn; 3 Department of Biology, College of Science, Imam Mohammad Ibn Saud Islamic University (IMSIU), Riyadh 11623, Saudi Arabia; nsbasher@imamu.edu.sa (N.S.B.); naabdalneim@imamu.eud.sa (N.A.I.); 4 College of Animal Science and Technology, Yangzhou University, Yangzhou 225009, China; mh23108@stu.yzu.edu.cn (S.Y.A.); elemlak1339@yzu.edu.cn (A.A.S.); 5 Animal and Fish Production Department, Faculty of Agriculture (Al-Shatby), Alexandria University, Alexandria City 11865, Egypt; 6 Jiangsu Co-Innovation Center for Prevention and Control of Important Animal Infectious Diseases and Zoonoses, Yangzhou 225009, China

**Keywords:** *Bacteroides thetaiotaomicron*, *E. coli*, probiotics, C57BL/6J mice, gut microbiota, short-chain fatty acid

## Abstract

Background/Objectives: *Enteropathogenic Escherichia coli* O157:H7 infection disrupts intestinal homeostasis, causing dysbiosis, barrier dysfunction, and inflammation. This study aimed to evaluate the protective efficacy and mechanisms of a novel probiotic, *Bacteroides thetaiotaomicron* type strain ATCC 29148, isolated from goat feces, against *E. coli* O157:H7-induced colitis. Methods: This study assessed the protective potential of the probiotic strain *Bacteroides thetaiotaomicron* *BT6* and *BT7* in vitro for GI tolerance, adhesion, and no adverse effects were observed. For the in vivo experiment, male C57BL/6J mice were divided into groups treated with *Bacteroides thetaiotaomicron* (*BT6*), PBS, *E. coli* O157:H7, or a combination. We employed integrated analyses including 16S rRNA gene sequencing, antioxidant status, cytokine profiling, and short-chain fatty acid (SCFA) measurement. Results: In vitro, *Bacteroides thetaiotaomicron* (*BT6* and *BT7*) showed high gastrointestinal tolerance (71.89–93.22% survival). In vivo, it significantly mitigated infection-associated weight loss and disease activity (*p* < 0.05). Probiotic treatment enhanced barrier integrity, reduced colonic inflammation, and modulated systemic immune responses, notably increasing anti-inflammatory IL-10 while decreasing pro-inflammatory cytokines TNF-α, IL-1β, and IL-6 (*p* < 0.05). It also alleviated oxidative stress by reducing malondialdehyde (MDA) and elevating antioxidant enzymes (SOD, CAT, GSH) and ATP. Fecal SCFA profiling revealed increased propionic and butyric acid. 16S sequencing indicated that *B. thetaiotaomicron* (*BT6*) administration increased beneficial families (*Lactobacillaceae*, *Muribaculaceae*) and suppressed pathobionts. Conclusions: *B. thetaiotaomicron* (*BT6*) probiotic with potential for mitigating enteropathogenic infection, an effect mainly determined by its capacity to reestablish the intestinal epithelial barrier and enhance global host health, and modulating the inflammatory response.

## Simple Summary

Infections with harmful bacteria like *E. coli* O157:H7 can severely disrupt the gut, causing inflammation and damage. This study explored whether administering specific beneficial bacteria, *Bacteroides thetaiotaomicron* (isolated from goat feces), could protect the gut. Using a mouse model of infection, we found that this probiotic treatment effectively reduced weight loss and disease severity. It helped restore a healthy balance of gut microbes, strengthened the gut’s protective barrier, and reduced harmful inflammation and oxidative stress. Our findings suggest that *Bacteroides thetaiotaomicron* (*BT6*) has strong potential as a therapeutic probiotic to combat intestinal infections and promote gut health.

## 1. Introduction

The gut microbiota, consisting of trillions of microorganisms, forms an interconnected ecological system that is crucial for host health. One of its key roles is to act as a biological barrier for the colonization of pathogenic bacteria within the intestines [1,2]. A shift into dysbiosis, a condition characterized by decreased microbial diversity and disrupted host–microbe communication, is currently widely linked to the development of many diseases. This relationship is particularly significant in the context of enteric infections, where pathogens such as *E. coli* O157:H7 can disrupt the gut microbiota and epithelial barrier. These two disorders differ in their patterns of inflammation: CD is characterized by patchy, transmural ulceration in sites anywhere in the gastrointestinal tract, whereas UC is characterized by continuous inflammation in only the colon and almost always affects the rectum [3].

In the face of mounting constraints on antibiotic inclusion in feed for food-producing animals, research has turned to the discovery of acceptable alternatives to antibiotics. Leading candidates for these alternatives include probiotics, prebiotics, supplemental enzymes, phytogenic extracts, organic acids, and antimicrobial peptides [4,5,6].

Research into physiological mechanisms indicates that probiotics maintain gut barrier integrity by modulating microbial communities. They can counteract the age-associated rise of intestinal permeability and the resulting systemic inflammation by decreasing both gut barrier leakage and local inflammatory reactions [7]. There is evidence that the benefits of probiotics go beyond good digestive health to include a lowered risk of some cancers. These effects, like modulating the inflammatory response and improving the metabolism process, are associated with the capacity of probiotics to preserve balance in the gut microbiome and reduce the proliferation of harmful bacteria [8,9,10]. Probiotics not only improve systemic biological function but also have significant impacts on mental health and skin health through the gut–brain axis. They have potential applications in the beauty industry for skin products targeted at individuals seeking to enhance their appearance [11,12].

Residing as a core member of the mammalian gut microbiome, *B. thetaiotaomicron* can constitute up to 6% of the total bacterial community, underscoring its ecological significance. Genomic analysis in 2003 highlighted its extensive glycolytic and metabolic capabilities, but also revealed intrinsic resistance mechanisms to antibiotics, particularly erythromycin and tetracycline. Crucially, evidence suggests these resistance genes are mobile and capable of horizontal transfer to other commensals, presenting a potential vector for the spread of antibiotic resistance within the gut [13]. Despite this risk, its substantial contribution to host nutrition and gut homeostasis positions *B. thetaiotaomicron* as a leading candidate for next-generation probiotic (NGP) development [14].

The organism’s ability to hydrolyze glycosidic linkages in complex polysaccharides such as pullulan, amylose, and amylopectin is facilitated by its rich arsenal of glycolytic enzymes [15,16]. The resulting metabolic output is vital for host health, yielding significant quantities of short-chain fatty acids (SCFAs) including acetate, propionate, and succinate alongside hydrogen sulfide (H2S), which are crucial mediators of gut homeostasis. Notably, murine studies have shown that *B. thetaiotaomicron* inhibits the proliferation of *Clostridioides difficile* in the context of dysbiosis caused by a pathogen [17,18]. This antimicrobial interaction is mediated by two main mechanisms: modulation of the bile acid profile of the gut and restoration of microbial homeostasis. Furthermore, *B. thetaiotaomicron* is able to increase the prebiotic effect of the diet, i.e., H_2_S in the colon is a metabolite whose role is context-dependent; while it is a normal part of gut metabolism, elevated levels are often associated with dysbiosis and inflammation. This metabolic activity not only benefits the host but also leads to an increase in the growth of beneficial probiotic genera, such as *Lactobacillus* and *Bifidobacterium*, thus providing a synergistic microbial environment [19,20].

*B. thetaiotaomicron* causes mutual cross-feeding in the gastrointestinal tract, which activates the production of the proline-rich protein Sprr2A, necessary for the maintenance of desmosomes. In addition, this bacterium has been found to enhance the mucin ratio, mucus-related gene expression, and goblet cell formation in mice [21]. It has immunomodulatory effects, enhances resistance to infections, and regulates enteroendocrine cells. However, due to overgrowth, *B. thetaiotaomicron* can compromise the intestinal barrier and contribute to colorectal cancer, while *B. thetaiotaomicron* translocation into the circulation can cause bacteremia. Despite its positive health impact, its use in both food technology and supplements needs to be closely monitored and restricted to the right scenarios, including personalized nutrition [19,20,22]. Conversely, low doses of *B. thetaiotaomicron* have been shown to be effective in limiting the colonization of the pathogens in vivo via modification of the gut microbiome. A pathotype of *E. coli* known as enterohemorrhagic *E. coli* (EHEC) is classified based on its O antigen. Among these, the serotype O157:H7 is particularly virulent and a causative agent of infectious diarrhea and hemorrhagic enteritis in persons of all ages, including individuals of both infantile and adult age [23].

This study was designed to assess the protective capacity of *Bacteroides thetaiotaomicron* against intestinal infection caused by *Escherichia coli* O157:H7 in mice. Our goals were to characterize the biological properties of *B. thetaiotaomicron* strains isolated from healthy goats in vitro and to study its health-promoting mechanisms, including reinforcement of the gut barrier, inhibition of harmful bacteria growth, and control of early safety indicators (e.g., tissue health, SCFA production, antioxidant activity, and inflammation), as well as their effects on microbiota composition and host growth performance. Characterizing the functional attributes of these isolates will contribute to building a strong case, ultimately revealing the development of *Bacteroides thetaiotaomicron* as a new generation probiotic for application in livestock production.

## 2. Materials and Methods

### 2.1. Isolation and Cultivation of Bacteroides thetaiotaomicron Strain Designations *BT6*/*BT7*

A total of 47 fecal samples were taken from healthy goats and initially enriched in Gifu Anaerobic Medium (GAM). After purification, two isolates, designated *BT6* and *BT7*, were selected for further characterization based on acid production profiles.

The enriched samples were then serially diluted with phosphate-buffered saline (PBS) between 10^1^ and 10^8^. Subsequently, 100 microliters of the most dilute sample were inoculated on Centres for Disease Control (CDC) Anaerobic Blood Agar plates containing 1% of vitamin K and 5% of defibrinated sheep blood. All bacterial cultures were propagated under strict anaerobic conditions using an anaerobic jar system (Oxoid™ AnaeroJar™, Thermo Scientific, Waltham, MA, USA). Anaerobiosis was generated using a gas-generating sachet (AnaeroGen™, Oxoid, Basingstoke, UK), which produces an atmosphere consisting of approximately 80–85% N_2_, 10–15% CO_2_, and <5% H_2_. Oxygen depletion was monitored using anaerobic indicator strips (Resazurin-based, Oxoid), which remained colorless throughout the incubation period, confirming sustained anaerobiosis. All culture media were pre-reduced in the anaerobic jar for at least 24 h prior to inoculation. Following anaerobic incubation, serial dilutions (10^−1^ to 10^−8^) of the enriched fecal samples were spread-plated onto CDC Anaerobic Blood Agar. Single colonies exhibiting distinct morphological characteristics, including differences in size, color, edge, and elevation, were individually selected and re-streaked onto fresh CDC Anaerobic Blood Agar plates. This purification procedure was repeated for three consecutive passages to ensure the isolation of pure, axenic cultures corresponding to each distinct colony morphotype. Following incubation under the above-mentioned conditions, bacterial isolates were selected for further identification and preliminary characterization, including key biochemical assays and morphological observations such as cell shape analysis and Gram staining.

### 2.2. Genomic DNA Extraction

Genomic DNA was extracted using the Bacterial Genomic DNA Extraction Kit (Bioengineering Co., Shanghai, China) according to the manufacturer’s instructions (Protocol V2.1, Product No. SK8255). Briefly, 1.5 mL of overnight bacterial culture was centrifuged at 10,000× *g* for 2 min. The cell pellet was resuspended in 200 µL of lysis buffer containing 20 µL of proteinase K. A preliminary heat lysis step (95 °C for 10 min) was performed prior to column-based purification to enhance cell wall disruption. No further modifications were made to the manufacturer’s protocol. DNA purity and concentration were assessed spectrophotometrically (NanoDrop 2000, Thermo Scientific) by measuring A_260_/A_280_ and A_260_/A_230_ ratios.

A 50 µL polymerase chain reaction (PCR) was prepared using a standard master mix and 10 pmol of each primer. The universal primers 27F (5′-AGAGTTTGATCCTGGCTCAG-3′) and 1492R (5′-GGTTACCTTGTTACGACTT-3′) were used to amplify the target region. Amplification was carried out in a PeQlab thermocycler (Waltham, MA, USA) with the following protocol: initial denaturation at 94 °C for 10 min; 35 cycles of denaturation at 94 °C for 1 min, annealing at 58 °C for 1 min, and extension at 72 °C for 1 min and 30 s; followed by a final extension at 72 °C for 8 min. The amplified products were then sequenced commercially by Macrogen Inc. (Seoul, Republic of Korea) using the Sanger sequencing platform with the same primers to ensure bidirectional coverage. Taxonomic identification was performed by comparing these consensus sequences against the NCBI GenBank database using the BLAST (version 2.13.0) algorithm. Species-level assignment was based on the following criteria: (1) ≥99% sequence identity with the closest validated type strain, (2) query coverage of ≥98%, and (3) an E-value of 0.0. Isolates demonstrating between 97% and 98.99% similarity were classified at the genus level only.

After sequencing, a BLAST nucleotide search was performed, and a phylogenetic tree was made using MEGA11 software (Kimura-2-parameter, neighbor-joining, 1000 bootstrap replicates) [24].

### 2.3. Acid and Bile Salt Tolerance

To test the resistance to low pH and bile salts, isolates were grown in Gifu Anaerobic Medium (GAM) broth. For each test, 10 mL of a 24-h bacterial culture was centrifuged for 5 min at 4000× *g*. The cell pellet obtained was resuspended in either acidic or neutral pH solutions. Bacterial growth was monitored by measuring optical density at 600 nm using a spectrophotometer (Eppendorf, Hamburg, Germany) to assess biomass accumulation. To quantitatively assess viability, samples were serially diluted and plated on GAM agar immediately after stress exposure. Colony-forming units per milliliter (CFU/mL) were counted after 48 h of anaerobic incubation. Viability was calculated by comparing CFU/mL before and after treatment using the following formula:

(1) Survival (%) = (OD _after treatment_/OD _before treatment_) × 100

Gastrointestinal transit survival was assessed using a model of simulated digestion. Two selected strains that had initial concentrations ranging from 1.4 × 10^9^ to 6.6 × 10^9^ CFU/mL were used in a two-phase assay. The gastric phase was simulated by adding pepsin to a final concentration of 5% (*w*/*v*), adjusting the pH to 2.5, and incubating the samples at 37 °C with 450× *g* agitation for 3 h. For the next phase, i.e., the intestinal phase, a mixture of bile salts (0.3% *w*/*v*) and pancreatin (0.1% *w*/*v*) was added, the pH was adjusted to 7.0, and the mixture was incubated under the same conditions for 7 h. Post-digestion, the samples were serially diluted, plated in triplicate on nutrient agar (NA), and incubated at 37 °C for 48 h. The rate of survival through the simulated gastrointestinal tract (GIT) was then calculated as follows:

(2) Survival (%) = (log CFU N_1_/log CFU N_0_) × 100

where N_0_ and N_1_ represent the bacterial counts before and after the simulated treatment, respectively.

### 2.4. Cell Culture

The Caco-2 human colon adenocarcinoma cell line was acquired from the Korea Cell Line Bank (Seoul, Republic of Korea). Cells were grown in Dulbecco’s Modified Eagle’s Medium (DMEM; Gibco, Grand Island, NY, USA) supplemented with 10% heat-inactivated fetal bovine serum (FBS; Gibco) and 1% antibiotic–antimycotic solution (Gibco). They were maintained at 37 °C in a humidified incubator with 5% CO_2_ and passaged routinely upon reaching 80% confluence.

#### 2.4.1. Cell Cytotoxicity Assay

An assessment of bacterial cytotoxicity was performed in vitro, following a procedure adapted from Mohanty et al. [25]. Cell viability and metabolic activity were determined using the MTT assay, a colorimetric method based on the reduction of the yellow tetrazolium salt MTT to purple formazan crystals by mitochondrial dehydrogenases in living cells. Briefly, Caco-2 cells in the exponential growth phase were plated in 96-well plates at a density of 3 × 10^4^ cells per well in 100 µL of culture medium and allowed to adhere for 24 h. The medium was then replaced with 100 µL of filter-sterilized bacterial supernatant from each isolate, standardized to a concentration of 1 × 10^7^ CFU/mL. Cells were exposed to this supernatant for either 8 or 24 h. After the treatment period, 100 µL of MTT solution was added to each well, and the plates were incubated for 2 h at 37 °C. The resulting formazan crystals were solubilized by adding 100 µL of dimethyl-sulfoxide (DMSO) to each well, and the absorbance was measured at 570 nm using a microplate reader.

(3) Viability (%) = (A_ sample − A_ balnk)/(A_ control − A_ blank) × 100

#### 2.4.2. Adhesion to Caco-2 Cell Line

The ability of the candidate probiotic strains to adhere to intestinal epithelial cells was tested using a Caco-2 cell model, with adjustments based on the protocol by Fonseca et al. [26]. Caco-2 cells were plated in 12-well plates at 1 × 10^5^ cells/mL in complete DMEM and incubated for 24 h at 37 °C. Meanwhile, bacterial strains were grown in GAM broth for 18 h at 37 °C, collected by centrifugation at 4000× *g* for 20 min, and washed twice with PBS. The pellet was then resuspended in antibiotic-free DMEM to a density of 1 × 10^8^ CFU/mL.

This bacterial suspension was applied to the washed cell monolayers and incubated for 2 h at 37 °C in a 5% CO_2_ atmosphere. Following incubation, non-adherent bacteria were removed by rinsing the wells three times with PBS. Adherent bacteria were recovered by lysing the Caco-2 cells with 0.1% (*v*/*v*) Triton X-100 in PBS, and viable counts were determined by plating serial dilutions. Adhesion was expressed as the percentage of bacteria from the initial inoculum that remained attached to the cell layer.

(4) Adhesion (%) = (CFU adhered/CFU inoculated) × 100

### 2.5. Auto-Aggregation and Cell Surface Hydrophobicity Assays

Auto-aggregation ability was assessed after 16–18 h of bacterial growth. Cultures were standardized to an optical density of 0.5 McFarland at 600 nm to evaluate self-aggregation, a trait associated with intestinal colonization and antipathogenic effects.

The auto-aggregation ability of bacterial strains was assessed following 16–18 h of cultivation. To evaluate this trait, closely associated with intestinal colonization and antipathogenic activity, bacterial cultures were standardized to an optical density of 0.5 McFarland at 600 nm. Auto-aggregation was expressed as a percentage calculated from the decrease in optical density using the following formula:

(5) Auto-aggregation (%) = 1 − (A_t_/A_0_) × 100

where A_0_ represents the initial optical density at 600 nm, and A_t_ denotes the optical density at the same wavelength after the incubation period.

Cell surface hydrophobicity was evaluated according to the protocol outlined by Khushboo et al. [24]. Briefly, bacterial cultures were harvested by centrifugation at 6000× *g* for 10 min, and the pellets were washed and resuspended in phosphate-buffered saline (PBS). The cell density was adjusted to approximately 1 × 10^8^ CFU/mL based on a predetermined standard curve relating optical density at 600 nm (OD600) to viable plate counts. The initial absorbance (A_0_) of this standardized suspension was recorded at 600 nm. Next, 1 mL of xylene (Merck, Darmstadt, Germany) was combined with 5 mL of the bacterial suspension. The mixture was vortexed for 2 min and incubated at 37 °C for 1 h to permit phase separation. After incubation, the absorbance of the aqueous phase (A_1_) was measured at the same wavelength. Hydrophobicity was calculated as follows:

(6) Hydrophobicity (%) = (1 − A_1_/A_0_) 100

### 2.6. Antipathogenic Activity Detection (Antimicrobial Susceptibility Test with Pathogenic E. coli O157:H7)

The antimicrobial activity of the *B. thetaiotaomicron* (*BT6* and *BT7*) strains was evaluated against the pathogenic bacterium *E. coli* O157:H7. First, *B. thetaiotaomicron* was inoculated into GAM broth and cultured under anaerobic conditions to obtain a fresh bacterial suspension. Subsequently, the antimicrobial susceptibility test was performed using NA solid medium [27], in accordance with conventional assays for determining the antagonistic activity of both *B. thetaiotaomicron* (*BT6* and *BT7*) against *E. coli* O157:H7.

### 2.7. Antibiotic Susceptibility Testing

The antibiotic susceptibility profile of *B. thetaiotaomicron* (*BT6* and *BT7*) was evaluated against a panel of commonly used antibiotics, including ampicillin, gentamicin, and ciprofloxacin, among others. The Kirby–Bauer disk diffusion method was employed in strict accordance with the guidelines provided by the Clinical and Laboratory Standards Institute (CLSI). After incubation, the area of inhibition around each antibiotic disk was measured using a caliper. These diameters were interpreted according to the guidelines of CLSI to classify the susceptibility of *B. thetaiotaomicron* (*BT6* and *BT7*) to each antibiotic as susceptible, intermediate, or resistant.

### 2.8. Hemolytic Activity Assay

The hemolyticity of the bacterial isolates was observed using the method previously described. Bacterial strains were streaked onto Columbia blood agar plates (supplemented with 5% defibrinated sheep blood) and incubated under aerobic conditions at 37 °C for 48 h [28]. Hemolytic patterns were categorized into three types based on the reaction around the colonies: (a) β-hemolysis: complete lysis of red blood cells, resulting in a clear, colorless zone surrounding the colony; (b) α-hemolysis: partial hemolysis, manifesting as a greenish or brownish halo around the colony; (c) γ-hemolysis: no hemolytic activity, with no visible change in the blood agar around the colony. Strains exhibiting γ-hemolysis were classified as non-hemolytic and deemed safe for potential probiotic applications.

### 2.9. Probiotic Intervention with B. thetaiotaomicron (BT6) in Mice Challenged with Escherichia coli O157:H7

#### 2.9.1. Animal Allocation and Treatment Protocol

Healthy 6-week-old male C57BL/6J mice, with an initial body weight of 23 ± 3 g, were obtained from the Experimental Animal Center of Yangzhou University. The experimental protocol received approval from the Animal Care and Use Committee at the College of Veterinary Medicine, Yangzhou University (Approval ID: SCXK-[Su]-2021-0013). Forty genetically similar male C57BL/6J mice were randomly assigned to one of four treatment groups (*n* = 10 per group): a control group (C) receiving phosphate-buffered saline (PBS), a group administered *B. thetaiotaomicron* (*BT6*), a group challenged with *E. coli* O157:H7 (E), and a co-administration group receiving both *B. thetaiotaomicron* (*BT6*) and *E. coli* O157:H7 (M).

Animals were housed in standard plastic cages for the 28-day study period under controlled environmental conditions, which included a 12-h light/dark cycle, a temperature maintained between 33 and 35 °C, and relative humidity of 53–57%. After a 3-day adaptation period, all groups had ad libitum access to water and feed throughout the experiment.

Group E (*E. coli* mono-colonization): Received a daily oral gavage of 200 µL *E. coli* O157:H7 suspension at 1 × 10^9^ CFU/mL, corresponding to 2 × 10^8^ CFU per dose.

Group *BT6* (*B. thetaiotaomicron* mono-colonization): Received a daily oral gavage of 200 µL *B. thetaiotaomicron* suspension at 1 × 10^9^ CFU/mL (2 × 10^8^ CFU per dose).

Group M (Co-colonization): Received a daily oral gavage of 200 µL of a 1:1 (*v*/*v*) mixture of *E. coli* O157:H7 and *B. thetaiotaomicron* (*BT6*). Each strain was individually adjusted to 1 × 10^9^ CFU/mL prior to mixing, so that each dose delivered 2 × 10^8^ CFU of *E. coli O157:H7* and 2 × 10^8^ CFU of *B. thetaiotaomicron*.

Group C (Control): Received 200 µL of sterile phosphate-buffered saline (PBS) daily to control for gavage-induced stress.

We based the number of animals per group on previously published murine probiotic intervention studies, which typically use 6–10 mice per group to evaluate protective activity against enteric pathogens, including *E. coli* O157:H7. Several studies using similar infection models and probiotic treatments have employed comparable sample sizes, demonstrating that this range is sufficient to detect biologically meaningful differences in clinical scores, bacterial colonization, and inflammatory markers [29].

**Figure 1:**
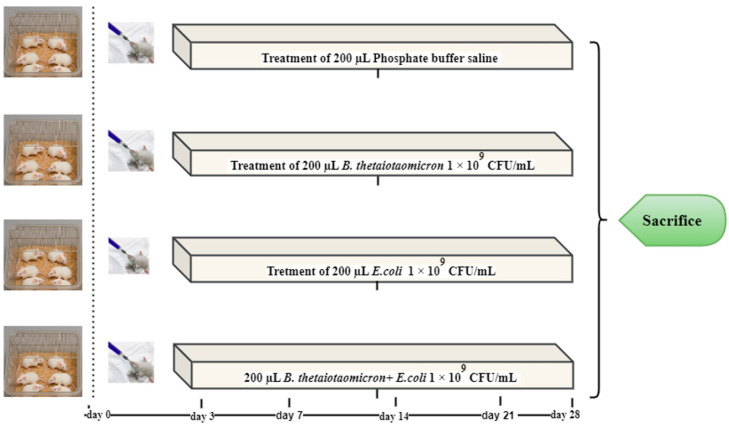
Animal experimental design for mice treated with probiotic and pathogenic bacteria.

The general condition of the mice was monitored three times daily, with records of abnormalities including mortality, diarrhea, lethargy, and dullness. Throughout the trial, each mouse was weighed, and its feed and water intake were quantified every two days, beginning on the first day of the experiment (Figure 1).

#### 2.9.2. Sample Collection

All experimental operations were performed under sterile conditions: gloves and surfaces were disinfected with 70% ethanol, and freshly excreted fecal pellets were collected using clean paper towels. *E. coli* O157:H7 stocks were first streaked onto LB agar plates and incubated at 37 °C for 24 h to obtain isolated colonies. Following this, individual colonies were inoculated into LB broth and grown for 12 h at 37 °C with constant agitation. *B. thetaiotaomicron* (*BT6*) was revived and inoculated on Bacteroides Bile Esculin (BBE) Agar. Single colonies were then selected for inoculation into GAM liquid medium and cultured at 37 °C for 24 h. The concentration of each strain was determined using the colony-forming unit (CFU) counting method. After 28 days of the experiment, the mice were fasted and weighed under uniform conditions. Final body weight (FBW), total feed intake, and total water intake were recorded accurately for each mouse.

#### 2.9.3. Disease Activity Index (DAI) and Colon Length

Clinical disease severity was measured on a standardized DAI for ulcerative colitis. During the infection phase, mice were monitored on a daily basis for changes in body weight, stool form, presence of diarrhea, and evidence of rectal bleeding. The final DAI score was the mean of 3 components: percentage weight loss, stool consistency, and fecal occult blood.

At the end of the experimental time, the mice were killed using cervical dislocation. The abdominal cavity was opened, and the whole colon was removed from the caecum to the anus. After light microscope examination with PBS, the colon length was measured and photographed. Tissue segments and luminal contents were promptly sited in cryovials, snap-frozen in liquid nitrogen, and transferred to a −80 °C freezer for subsequent analysis.

#### 2.9.4. Histological Analysis of Ileum Tissue

A 0.5 cm section of the mid-ileum was removed, washed with normal saline to re-rinse remnants of the contents, and stored in 4% paraformaldehyde for 24 h. Tissues were then embedded in paraffin, sectioned at 5 um, and deparaffinized in xylene. The sections were then rehydrated by means of a descending ethanol series. Finally, routine hematoxylin and eosin (H&E) staining was performed following established laboratory protocols.

The extent of ileum tissue damage was graded using a standardized scoring system for small intestine histopathology (0–5 points) based on the following criteria [30]: (a) score 0: normal colonic mucosa with intact crypts and surface epithelium; (b) score 1: mild crypt distortion and minimal inflammatory cell infiltration; (c) score 2: significant crypt hyperplasia and goblet cell depletion with moderate inflammatory infiltration; (d) score 3: severe crypt damage with erosion of the surface epithelium; (e) score 4: widespread epithelial ulceration and severe transmural inflammation; (f) score 5: complete loss of crypt architecture with extensive ulceration and inflammatory infiltrates.

### 2.10. Measurement of Serum Inflammatory Cytokines

Serum levels of specific cytokines were measured using commercial enzyme-linked immunosorbent assay (ELISA) kits according to the manufacturers’ protocols. Blood samples were collected from the mice via puncture of the caudal vena cava, and serum was isolated through centrifugation. The concentrations of selected inflammatory mediators’ interleukin-1β (IL-1β), interleukin-6 (IL-6), tumor necrosis factor-α (TNF-α), and the anti-inflammatory cytokine interleukin-10 (IL-10), were quantified in the prepared serum samples. All experimental procedures were performed in full compliance with the protocols provided by the kit manufacturers.

### 2.11. Antioxidant Capacity Analysis

The antioxidant status of colon tissue was evaluated by measuring the levels of key oxidative stress markers and antioxidant enzymes; the measured parameters included malondialdehyde (MDA) concentration and the activities of catalase (CAT), glutathione-peroxidase (GSH-Px), and superoxide dismutase (SOD).

All analyses were performed spectrophotometrically using a PU 8720 UV/VIS scanning spectrophotometer. Commercial assay kits (Nanjing Jiancheng Bioengineering Institute, Nanjing, China) were used for each parameter, and all experimental procedures were conducted in strict accordance with the manufacturer’s protocols.

### 2.12. Reactive Oxygen Species (ROS) and ATPase Activity

Intracellular ROS in ileal tissue were quantified using a commercial fluorometric assay (OxySelect™ In Vitro ROS/RNS Assay Kit, Cell Biolabs, San Diego, CA, USA, STA-347). Briefly, tissue homogenates were prepared in cold phosphate-buffered saline (PBS) and centrifuged; the resulting supernatant was used for the assay. This method relies on the oxidation of the non-fluorescent substrate DCFH-DiOxyQ to the fluorescent compound DCF in the presence of ROS. Fluorescence was recorded with excitation at 480 nm and emission at 530 nm. Separately, adenosine triphosphate (ATP) levels in colon tissue were determined with a bioluminescence-based ATP assay kit (Beyotime, Shanghai, China, S0026), according to the manufacturer’s protocol.

### 2.13. Short-Chain Fatty Acid Analysis

The concentrations of short-chain fatty acids (SCFAs), including acetic acid, propionic acid, isobutyric acid, butyric acid, isovaleric acid, pentanoic acid, and hexanoic acid, were quantified in mouse fecal samples using liquid chromatography. Approximately 50 mg of fresh fecal samples was mixed with 500 µL of ultrapure water, sonicated for 10 min at 25 °C to facilitate extraction, and then spiked with 50 µL of internal standard solution (2-ethylbutyric acid, 100 µg/mL). After vigorous vortexing for 2 min and centrifugation at 12,000× *g* for 10 min at 4 °C, 200 µL of the supernatant was collected for derivatization.

Chemical derivatization was performed by adding 100 µL of 200 mM 3-nitrophenylhydrazine (3-NPH) in methanol and 100 µL of 120 mM 1-ethyl-3-(3-dimethylaminopropyl) carbodiimide (EDC) in methanol containing 6% pyridine. The mixture was incubated at 40 °C for 30 min with shaking at 500 rpm. Following derivatization, the samples were diluted with 200 µL of methanol:water (1:1, *v*/*v*) containing 0.1% butylated hydroxytoluene (BHT) to prevent oxidation, and centrifuged at 12,000× *g* for 5 min to clarify the supernatant.

Analysis of the derivatized extracts was carried out using ultra-performance liquid chromatography-mass spectrometry (UPLC-MS) on a Waters Acquity UPLC system coupled to a Xevo TQ-S mass spectrometer (Xevo, Bellevue, WA, USA). Chromatographic separation was achieved on a Waters UPLC BEH C8 column (100 mm × 2.1 mm, 1.7 µm particle size) maintained at 40 °C. The mobile phase consisted of (A) water with 0.01% formic acid and (B) acetonitrile with 0.01% formic acid, at a flow rate of 0.4 mL/min. The gradient program was as follows: 0–1 min, 25% B; 1–5 min, 25–60% B; 5–7 min, 60–100% B; 7–9 min, 100% B; 9–9.5 min, 100–25% B; and 9.5–12 min, 25% B for re-equilibration. The injection volume was 2 µL. Mass spectrometry was performed in negative electrospray ionization mode with multiple reaction monitoring (MRM) for each derivatized SCFA. Quantification was achieved by comparing peak areas with calibration curves constructed using authentic standards.

### 2.14. Extraction of Bacterial Genomic DNA and 16S rDNA Sequencing Analysis

Total genomic DNA was isolated from mouse fecal samples with a commercial extraction kit (TianGen Biochemical Technology Co., Ltd., Beijing, China), following the provided protocol. This DNA served as the template for polymerase chain reaction (PCR) amplification of the V3–V4 hypervariable regions of the bacterial 16S rRNA gene, using the universal primer pair 341F (5′-CCTACGGGNGGCWGCAG-3′) and 806R (5′-GGACTACHVGGGTATCTAAT-3′) [31]. The resulting PCR amplicons were purified with a commercial DNA clean-up kit (Universal DNA Purification Kit, TianGen). Sequencing libraries were then constructed from the purified products using the NEBNext^®^ Ultra DNA Library Prep Kit (New England Biolabs, Inc., Beijing, China) according to the manufacturer’s instructions. Library quality was assessed on an Agilent 2100 Bioanalyzer (Agilent Technologies, Santa Clara, CA, USA). Sequencing was performed on an Illumina NovaSeq 6000 platform (Illumina, San Diego, CA, USA) using paired-end sequencing with 2 × 250 bp read length. A sequencing depth of approximately 50,000 raw reads per sample was targeted. The raw sequencing data have been deposited in the NCBI Sequence Read Archive under BioProject accession PRJNA753235.

### 2.15. Bioinformatics and Statistical Analysis

Raw sequencing data were processed using a unified workflow implemented in QIIME2 (version 2023.5). Demultiplexed paired-end reads were first quality-filtered, denoised, and merged using the DADA2 plugin within QIIME2. The following parameters were applied: truncation positions were set to 240 bp for forward reads and 210 bp for reverse reads based on quality score visualization, and reads with ambiguous bases or exceeding maximum expected errors (2) were discarded. This approach resolves amplicon sequence variants (ASVs) at single-nucleotide resolution, replacing the traditional 97% OTU clustering method [32,33].

For taxonomic assignment, representative ASV sequences were classified using a pre-trained Naive Bayes classifier (the RDP classifier algorithm implemented in QIIME2) against the SILVA reference database (version 138) [34]. A minimum confidence threshold of 70% was applied for taxonomic assignment. The Greengenes2 database was used for confirmatory comparisons where indicated [35].

Alpha diversity metrics were calculated to assess within-sample diversity. Specifically, we computed: (1) Chao1 index for community richness, (2) Shannon diversity index for species evenness and richness, (3) Simpson index for dominance, (4) Pielou’s evenness index, and (5) Faith’s phylogenetic diversity (PD) index. Rarefaction curves were generated to verify sequencing depth adequacy. Differences in alpha diversity between groups were evaluated using the Kruskal-Wallis test followed by pairwise Wilcoxon tests with Benjamini-Hochberg false discovery rate (FDR) correction.

Beta diversity analysis was performed to compare microbial community structure between groups. Principal coordinate analysis (PCoA) was conducted based on Bray-Curtis dissimilarity (abundance-based) and unweighted UniFrac distances (phylogeny-based). Permutational multivariate analysis of variance (PERMANOVA) with 999 permutations was used to test for significant differences in community composition among experimental groups, implemented in the vegan package (version 2.6–4) in R (version 4.2.1). For pairwise comparisons between groups, PERMANOVA was performed with 999 permutations per comparison, and *p*-values were adjusted for multiple testing using the Benjamini-Hochberg false discovery rate (FDR) correction. Statistical significance was set at adjusted *p* < 0.05.

Differential abundance analysis of specific taxa was performed using linear discriminant analysis effect size (LEfSe) with the following parameters: alpha value for Kruskal-Wallis test < 0.05, and logarithmic LDA score threshold > 2.0 [36,37].

Functional profiling of microbial communities was predicted using PICRUSt2 (Phylogenetic Investigation of Communities by Reconstruction of Unobserved States, version 2.5.2) [38]. Predicted metagenomes were mapped to the Kyoto Encyclopedia of Genes and Genomes (KEGG) database (release 2023) to infer potential functional pathway alterations. Differential functional pathway abundance between groups was assessed using the STAMP software package (version 2.1.3) with Fisher’s exact test and Benjamini-Hochberg correction.

All raw 16S rRNA gene sequences generated in this study have been deposited in the NCBI Sequence Read Archive under BioProject accession PRJNA753235. Visualizations were generated using the ggplot2 package in R [36].

### 2.16. Statistical Analysis

Data were analyzed using IBM SPSS Statistics (Version 26.0, IBM Corp., Armonk, NY, USA) and GraphPad Prism (Version 9.0, GraphPad Software, San Diego, CA, USA). For parameters measured repeatedly over time on the same animals, including body weight, feed intake, and water intake, a linear mixed model (LMM) for repeated measures was employed. The model included treatment group (C, BT, E, and M), time (day of measurement), and their interaction as fixed effects, with mouse subjects included as a random effect to account for within-subject correlation. An autoregressive (AR1) covariance structure was selected based on the lowest Akaike Information Criterion (AIC) value.

For data collected at a single time point (e.g., organ weights, colon length, cytokine concentrations, antioxidant parameters, SCFA levels, and alpha diversity indices), one-way analysis of variance (ANOVA) was performed. When significant differences were detected (*p* < 0.05), Tukey’s honest significant difference (HSD) post hoc test was applied for multiple pairwise comparisons between treatment groups.

For non-normally distributed data (assessed by Shapiro-Wilk test), the Kruskal-Wallis test followed by Dunn’s post hoc test with Bonferroni correction was used. All data are presented as mean ± standard deviation (SD). Statistical significance was defined as *p* < 0.05. Significance levels in figures are denoted as follows: * *p* < 0.05, ** *p* < 0.01, and *** *p* < 0.001.

## 3. Results

### 3.1. Isolation and Identification of B. thetaiotaomicron Strains Derived from Healthy Goats

**Figure 2:**
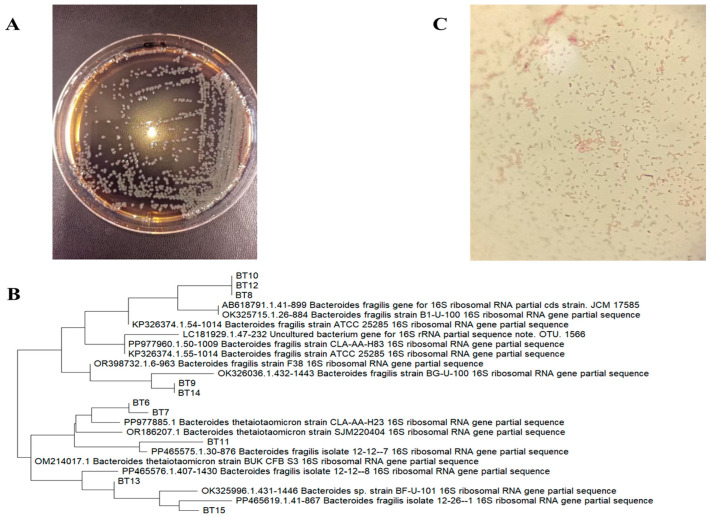
(**A**) Colonial morphology of Bacteroides isolates grown on Bacteroides Bile Esculin Agar. (**B**) Evolutionary relationships inferred from 16S rDNA sequence alignment, depicting both the obtained isolates and relevant reference strains. (**C**) Gram stain results under a microscope.

Both candidate *B. thetaiotaomicron* strains (*BT6* and *BT7*) were positive for esculin hydrolysis. Colonies grown on BBE Agar appeared gray to gray-white, with a blackened zone surrounding each colony (Figure 2A). Based on this characteristic, 47 strains showing distinct black halos were selected, purified, cultured, and preserved. Among these, 15 strains with the most pronounced acidification ability were chosen for further acid production performance evaluation and designated as BT1–BT15 (Figure 2B). The acid production curves of these 15 strains (Figure S1) indicate a rapid pH decline during the initial 0–12 h of fermentation, followed by a stable phase after 18 h. Strains *BT6* and *BT7* exhibited the highest and most consistent acid production, and were therefore selected for subsequent experiments.

Gram staining and microscopic observation revealed that *BT6* and *BT7* were Gram-negative bacilli (Figure 2C). Carbohydrate fermentation profiles (Table S1) revealed that the two isolates utilized cellobiose, maltose, mannitol, salicin, sucrose, raffinose, lactose, and glucose, whereas no growth was observed on sorbitol or inulin. They tested positive for catalase activity, the methyl red reaction, the Voges–Proskauer test, citrate utilization, nitrate reduction, amylase hydrolysis, motility, and indole production. These phenotypic traits are consistent with beneficial probiotic strains, reflecting their adaptability to the gastrointestinal environment and potential to regulate microbial ecology.

PCR amplification of the 16S rRNA gene yielded distinct, single bands when separated by 1.2% agarose gel electrophoresis. The amplified fragment size was approximately 1500 base pairs, which is consistent with the expected design (Figure S2). Homology analysis revealed 98% similarity between strains *BT6* and *BT7* (Figure 2B). Comparative sequence analysis against the NCBI GenBank database using the BLASTn algorithm demonstrated that both isolates shared 99.7% (*BT6*) and 99.5% (*BT7*) sequence identity with the *Bacteroides thetaiotaomicron* type strain ATCC 29148. Based on morphological, physiological, and biochemical characteristics, together with 16S rDNA sequence analysis, both strains were conclusively identified as *Bacteroides thetaiotaomicron*.

### 3.2. In Vitro Probiotic B. thetaiotaomicron Property Tests

#### 3.2.1. Extreme Heat Tolerance, pH, and Bile Salt Tolerance Test

**Figure 3:**
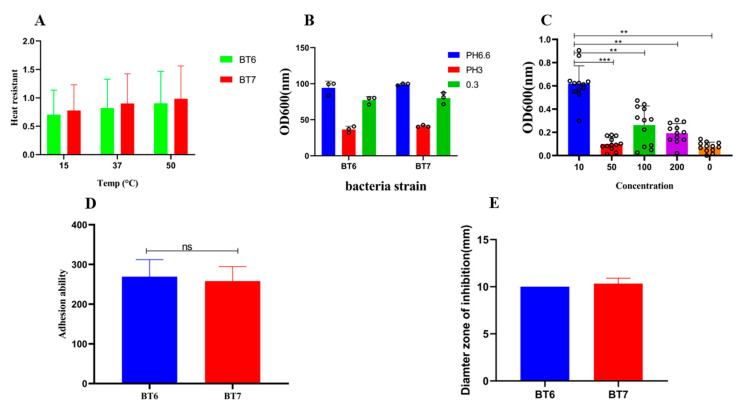
Phenotypic characterization of two bacterial strains, *BT6* and *BT7*. (**A**) Heat resistance of strains *BT6* (green bars) and *BT7* (red bars) after exposure to different temperatures (15 °C, 37 °C, and 50 °C). (**B**) Growth of strains *BT6* and *BT7* in media at different pH levels (pH 6.0, pH 3.0, and 0.3), measured as optical density at 600 nm (OD600). Green bars indicate pH 6.0 and red bars indicate pH 3.0 for both strains. (**C**) Effect of different concentrations (10, 50, 100, 200, and 0 μg/mL) on the growth (OD600) of the two strains. (**D**) Adhesion ability of strains *BT6* (blue bar) and *BT7* (red bar) to a surface or cell line. (**E**) Diameter of the inhibition zone (mm) produced by strains *BT6* (blue bar) and *BT7* (red bar) against a test microorganism. Statistical significance is indicated by asterisks (** *p* < 0.01, *** *p* < 0.001). “ns” indicates no statistically significant difference between the two strains.

The survival rate of *B. thetaiotaomicron* (*BT6* and *BT7*) was greater than 60% at 50 °C, 57% at 37 °C for 10 min, and 35% at 15 °C (Figure 3A), suggesting the ability of the strains to survive at high and low temperatures. The two selected isolates were able to survive for 2 h in pH 3.0 (acidic) and pH 6.6 (neutral) (Figure 3B). For these pH values, the survival rates were similar; interestingly, all the tested isolates had a survival rate greater than 60% when incubated in a low concentration of bile salts for 2 h.

#### 3.2.2. In Vitro Gut Transit Simulation

To assess gastrointestinal tolerance, the isolates were exposed to a simulated digestive environment, covering 3 h of exposure to artificial gastric juice and 7 h of incubation in artificial intestinal juice. The results (Table 1) reveal strong survival under these stressful conditions overall. All the isolates showed high levels of resistance to gastric juice, and their survival rates were between 78.69 and 97.65%. The two selected strains (*BT6* and *BT7*) tested especially well in intestinal juice, where 71.36% and 93.26% survival rates were achieved, respectively. These findings support the idea that the strains possess potential properties that allow them to survive passage through the murine gastrointestinal tract.

#### 3.2.3. Auto-Aggregation and Cell Surface Hydrophobicity

The auto-aggregation capacity of both strains was assessed using the optical density method. Briefly, bacterial suspensions were adjusted to an initial OD600 of approximately 0.5 and allowed to stand undisturbed. The decrease in OD600 over time, due to settling of aggregated cells, was monitored. A higher final OD600 relative to initial OD600 indicates less aggregation. Throughout the 24-h incubation period, both strains showed consistently low auto-aggregation, with OD ratios remaining near 1.0, indicating minimal settling of bacterial cells. At 24 h, the OD ratio for *BT6* was 1.07 and for *BT7* was 0.91 (Table 2), suggesting that these strains do not exhibit strong auto-aggregation under the tested conditions. It is important to note that OD600 in this assay serves as a proxy for cell sedimentation rate and does not measure bacterial viability. Additionally, the two isolates exhibited high cell surface hydrophobicity, ranging from 61% to 93% (Table 2). In vitro antagonistic tests demonstrated that the supernatant of *B. thetaiotaomicron* had a significant inhibitory effect on pathogenic *E. coli* O157:H7 (*p* < 0.01).

### 3.3. Antibiotic Susceptibility Profiles and Hemolytic Activity

Antimicrobial susceptibility testing showed that both tow strains of *B. thetaiotaomicron* (*BT6* and *BT7*) were susceptible to ten commonly used antibiotics, with intermediate susceptibility to gentamicin and amikacin (Table 3). Hemolytic activity assessment on Columbia blood agar plates revealed that 13 out of 15 isolates exhibited α-hemolysis or β-hemolysis. In contrast, strains *BT6* and *BT7* showed γ-hemolysis (non-hemolytic activity), suggesting they lack this particular virulence factor and are considered safe based on this preliminary criterion, although a comprehensive safety assessment would require additional testing (Table S2).

### 3.4. Cell Cytotoxicity Assay Analysis

Adhesion to intestinal epithelial cells is a crucial trait for probiotics. Prior to adhesion testing, the cytotoxicity of the probiotic strains was evaluated in Caco-2 cells to ensure no adverse effects were observed. Even at concentrations up to 1 × 10^9^ CFU/mL, the *BT6* strain showed no cytotoxic effects (Figure 3C). When tested at a concentration of 1 × 10^8^ CFU/mL, the percentages of *BT6* and *BT7* adhering to Caco-2 monolayers were 85.2 ± 5.2% and 71.2 ± 5.4%, respectively. The adhesion capacity did not differ significantly between the two isolates (Figure 3D).

### 3.5. B. thetaiotaomicron Alleviates E. coli- O157:H7-Induced Diarrhea in Mice

**Figure 4:**
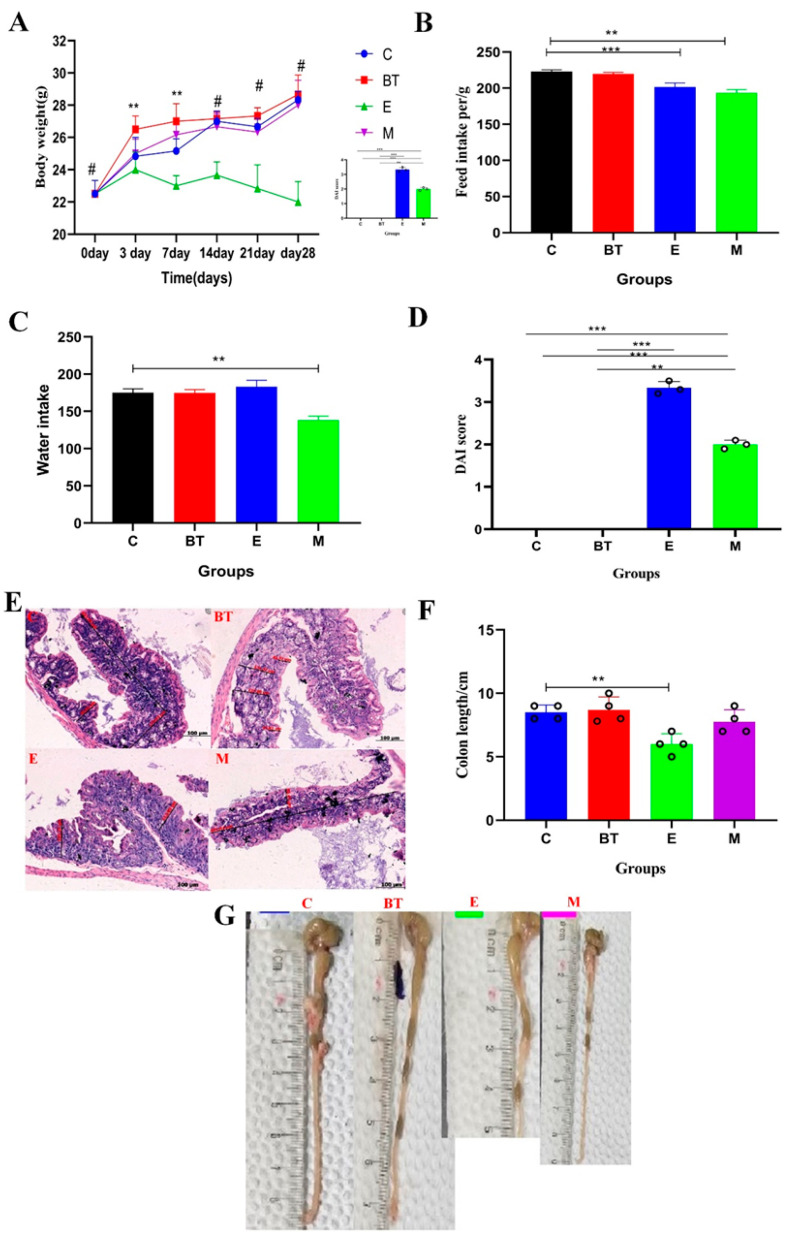
Effects of B. thetaiotaomicron (*BT6*) and its metabolites in *E. coli* O157:H7-infected mice. Evaluation of (**A**) body weight change, (**B**) food intake, (**C**) water consumption, (**D**) disease activity index (DAI) scores, (**E**) representative hematoxylin and eosin (H&E)-stained histological sections of colon tissue images show mucosal architecture, inflammation, and tissue damage at different magnifications or regions, and (**F**,**G**) colon length. Data are presented as the mean ± standard deviation. Statistical significance is denoted as follows: # indicates non-significance, ** *p* < 0.01, and *** *p* < 0.001. The sample size (n) for each experimental group is provided in the figure legends.

Administration of *B. thetaiotaomicron* (*BT6*) effectively alleviated *E. coli* O157:H7-induced diarrhea in mice and mitigated the pathogen’s adverse effects on growth performance. Mice infected with *E. coli* O157:H7 (E group) exhibited significantly lower body weight compared to the control group (C group) on days 7, 14, 21, and 28 (*p* < 0.01). However, supplementation with *BT6* significantly improved body weight in the treated groups relative to the *E. coli*-infected group (Figure 4A).

Mice supplemented with the probiotic showed a significant increase in feed intake relative to the infected group (*p* < 0.05), although the control group consistently consumed the most feed throughout the study (Figure 4B). Water intake was significantly reduced in *B. thetaiotaomicron* (*BT6*) + *E. coli* O157:H7 combination group (M group) compared to the control group (*p* < 0.05; Figure 4C). While reduced water intake may appear counterintuitive in an infectious diarrhea model, this finding is discussed in context below.

The disease activity index (DAI) was significantly elevated in the E group compared to the control (*p* < 0.05). Both the E group and M group showed markedly higher DAI scores than the *BT6* group (*p* < 0.05; Figure 4D), indicating that *BT6* supplementation effectively mitigated disease severity.

Infection with *E. coli* O157:H7 resulted in a significant reduction in colon length relative to the control group (*p* < 0.05; Figure 4F). Hematoxylin and eosin (H&E) staining of ileum tissue sections further confirmed the protective effect of *BT6*: the E group showed obvious intestinal mucosal damage, while the *BT6* group and M group exhibited reduced inflammatory cell infiltration and improved tissue integrity (Figure 4E).

Collectively, these results demonstrate that *B. thetaiotaomicron* (*BT6*) supplementation can ameliorate multiple physiological and morphological abnormalities associated with *E. coli* O157:H7 infection in mice, including growth retardation, abnormal water intake, increased disease severity, and intestinal structural damage.

### 3.6. Histopathological Changes in the Ileum

**Figure 5:**
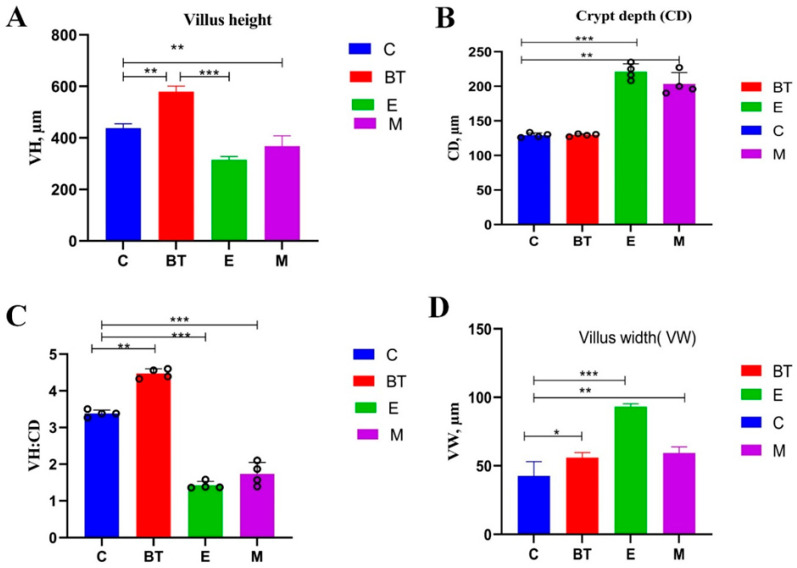
The effects of *B. thetaiotaomicron* (*BT6*) on ileum histomorphology in C57BL/6J mice infected with *E. coli* O157:H7. (**A**) Crypt depth (CD), (**B**) mucosal thickness, (**C**) crypt density, and (**D**) quantification of small intestine (ileum) epithelial integrity. Data are expressed as mean ± SD. Asterisks denote statistical significance (* *p* < 0.05, ** *p* < 0.01, *** *p* < 0.001).

Histomorphological analysis of the ileum showed significant differences among experimental groups (Figure 5). There was a significant decrease in crypt depth and mucosal thickness in the *E*. *coli* O157:H7-infected (E) and co-administration (M) groups compared to the C group (*p* < 0.05; Figure 5A,C). In contrast, treatment with *B. thetaiotaomicron* (*BT6* group) improved these morphological parameters compared to the infected group (*p* < 0.05), indicating restoration of normal ileum architecture.

Regarding crypt depth (CD) and mucosal width, the E group and M group showed significant alterations compared to the C group (*p* < 0.05; Figure 5B,D). Notably, supplementation with *B. thetaiotaomicron* (*BT6*) reversed these pathological changes: the *BT6* group displayed significantly improved crypt architecture relative to the E group (*p* < 0.05), approaching the morphology observed in the C group. No such corrective effects were seen in the C group, as it maintained normal ileum histomorphology throughout the experiment.

### 3.7. Inflammatory Markers

**Figure 6:**
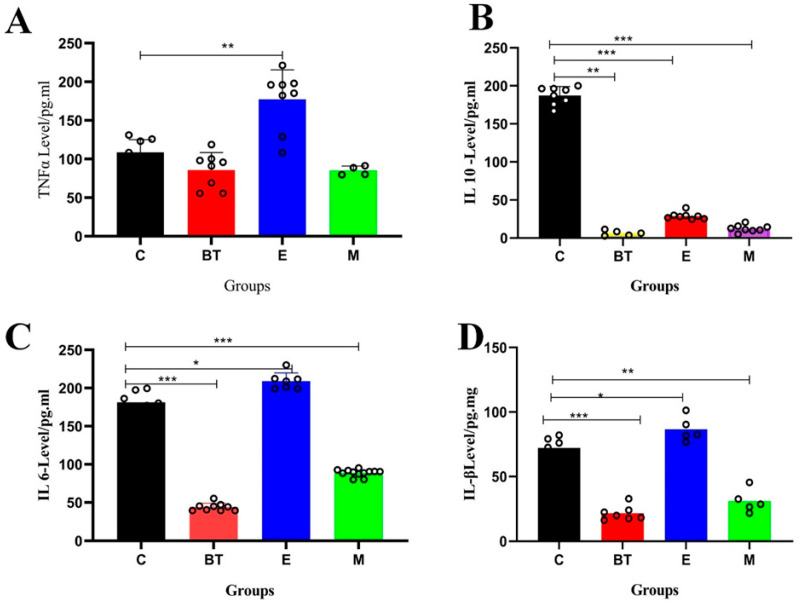
Modulation of systemic cytokine levels by *B. thetaiotaomicron* (*BT6*) in mice challenged with *E. coli* O157:H7. Serum concentrations of (**A**) TNF-α, (**B**) IL-10, (**C**) IL-6, and (**D**) IL-1β are shown as mean ± SD. Statistical analysis was performed using one-way ANOVA with Tukey’s post hoc test (* *p* < 0.05, ** *p* < 0.01, *** *p* < 0.001).

To further evaluate systemic inflammatory responses associated with colon pathology, serum cytokine levels were measured. As illustrated in Figure 6, infection with *E. coli* O157:H7 (E group) resulted in significantly higher serum levels of the pro-inflammatory mediators IL-1β, IL-6, and TNF-α, coupled with a pronounced decrease in the anti-inflammatory cytokine IL-10, when compared to the control (C) group (*p* < 0.05; Figure 6A–D). Treatment with *B. thetaiotaomicron* (*BT6* group) effectively reversed these changes: pro-inflammatory cytokine levels were significantly lowered, while IL-10 levels were substantially elevated (*p* < 0.05). These results confirm that *B. thetaiotaomicron* modulates the inflammatory response to restore immune balance, which correlates with the improvement in colon histopathological changes observed in the BT group.

### 3.8. Assessment of Antioxidant Capacity

**Figure 7:**
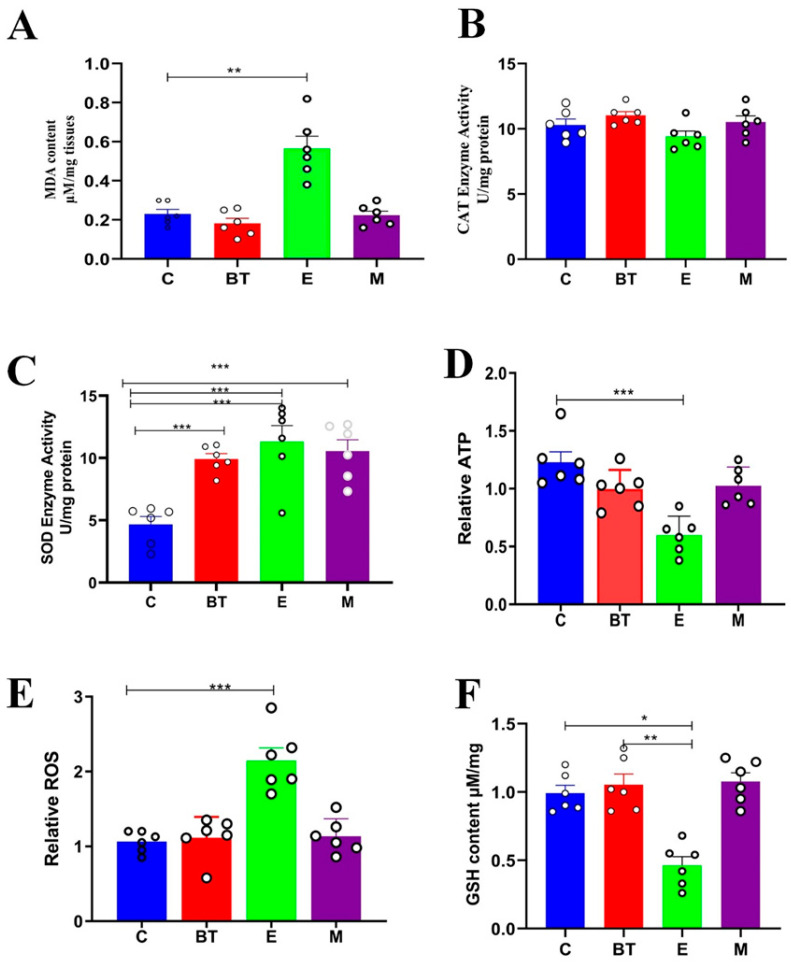
The effects of the *B. thetaiotaomicron* (*BT6*) probiotic in C57BL/6J mice infected with *E. coli* on oxidative stress were examined. (**A**) Levels of malondialdehyde (MDA), a marker of lipid peroxidation, (**B**) activity of the antioxidant enzyme catalase (CAT), (**C**) activity of superoxide dismutase (SOD), (**D**) ATP levels, (**E**) reactive oxygen species (ROS) levels, and (**F**) activity of glutathione peroxidase (GSH-Px). The data are presented as the means ± standard deviation. * *p* < 0.05, ** *p* < 0.01 and *** *p* < 0.001, where n represents the number of replications.

Infection with *E. coli* O157:H7 significantly increased oxidative stress in colon tissue. Relative to the uninfected control group (C group), MDA and ROS concentrations were significantly higher in mice in the infected group (E), as were the activities of the antioxidant enzymes SOD and CAT (Figure 7A–C,E). This pattern represents a strong compensatory response to oxidative injury. In contrast, levels of reduced GSH, a major cellular antioxidant, were significantly decreased in the infected group compared to controls (*p* < 0.05; Figure 7F).

Additionally, colonic ATP content was significantly elevated in the *E. coli*-infected group relative to the control group (*p* < 0.05; Figure 7D), reflecting altered energy metabolism in response to pathogenic infection. Notably, administration of the probiotic *B. thetaiotaomicron* (*BT6* group) significantly restored all these oxidative stress-related parameters toward normal levels (*p* < 0.05). These results indicate that *B. thetaiotaomicron* (*BT6*) plays a critical role in mitigating oxidative damage and enhancing the host’s antioxidant defense mechanisms during *E. coli* O157:H7-induced enteric infection.

### 3.9. Fecal Short-Chain Fatty Acid Profiles

Profiling of fecal SCFAs demonstrated marked variations in the concentrations of principal fermentation products among the experimental groups, reflecting the regulatory effect of *B. thetaiotaomicron* (*BT6*) on gut microbial metabolism during *E. coli* O157:H7 infection.

Fecal concentrations of pentanoic (valeric) acid, butyric acid, and isovaleric acid were significantly elevated in mice treated solely with *B. thetaiotaomicron* (BT group) when compared to those infected with *E. coli* (E group) or receiving the combined bacterial challenge (M group) (*p* < 0.05). Propionic acid and isobutyric acid levels were notably elevated in the M group relative to the control group (C group) (*p* < 0.05). Hexanoic (caproic) acid content decreased across all groups except the M group, though this change did not reach statistical significance (*p* > 0.05).

**Figure 8:**
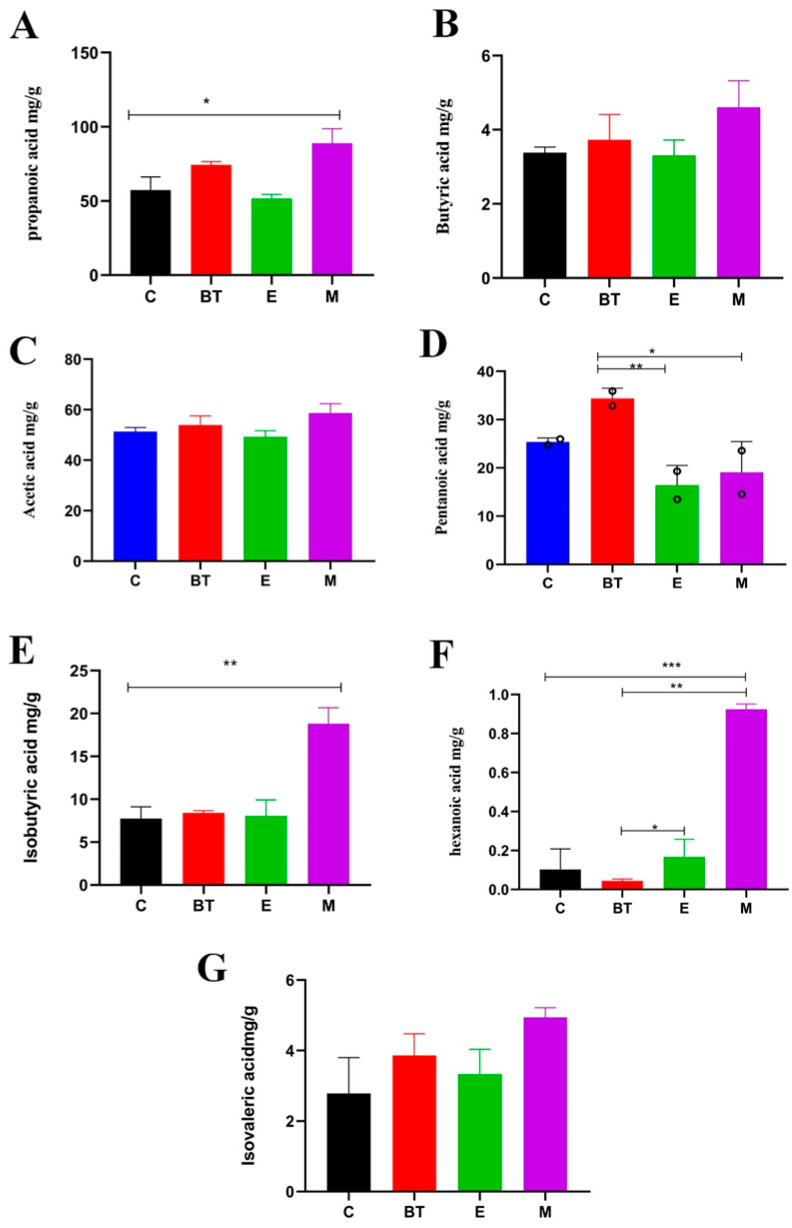
*B. thetaiotaomicron* (*BT6*) modulates short-chain fatty acid (SCFA) production during *E. coli* O157:H7 infection. Fecal SCFA concentrations were measured across experimental groups: (**A**) acetic acid, (**B**) propionic acid, (**C**) butyric acid, (**D**) pentanoic acid, (**E**) isobutyric acid, (**F**) hexanoic acid, (**G**) isovaleric acid. Values represent mean ± standard deviation. Significance levels: * *p* < 0.05, ** *p* < 0.01, *** *p* < 0.001.

While levels of acetic, butyric, and isovaleric acids appeared to recover in the BT group relative to the E group, statistical comparison across all four experimental groups did not reveal significant differences for these particular SCFAs (Figure 8A–G). Collectively, the data suggest that *B. thetaiotaomicron* (*BT6*) influences the gut microbial metabolome by shifting the production of specific short-chain fatty acids, a change that likely supports the re-establishment of intestinal homeostasis following infection with *E. coli* O157:H7.

### 3.10. Sequencing Data Analysis

**Figure 9:**
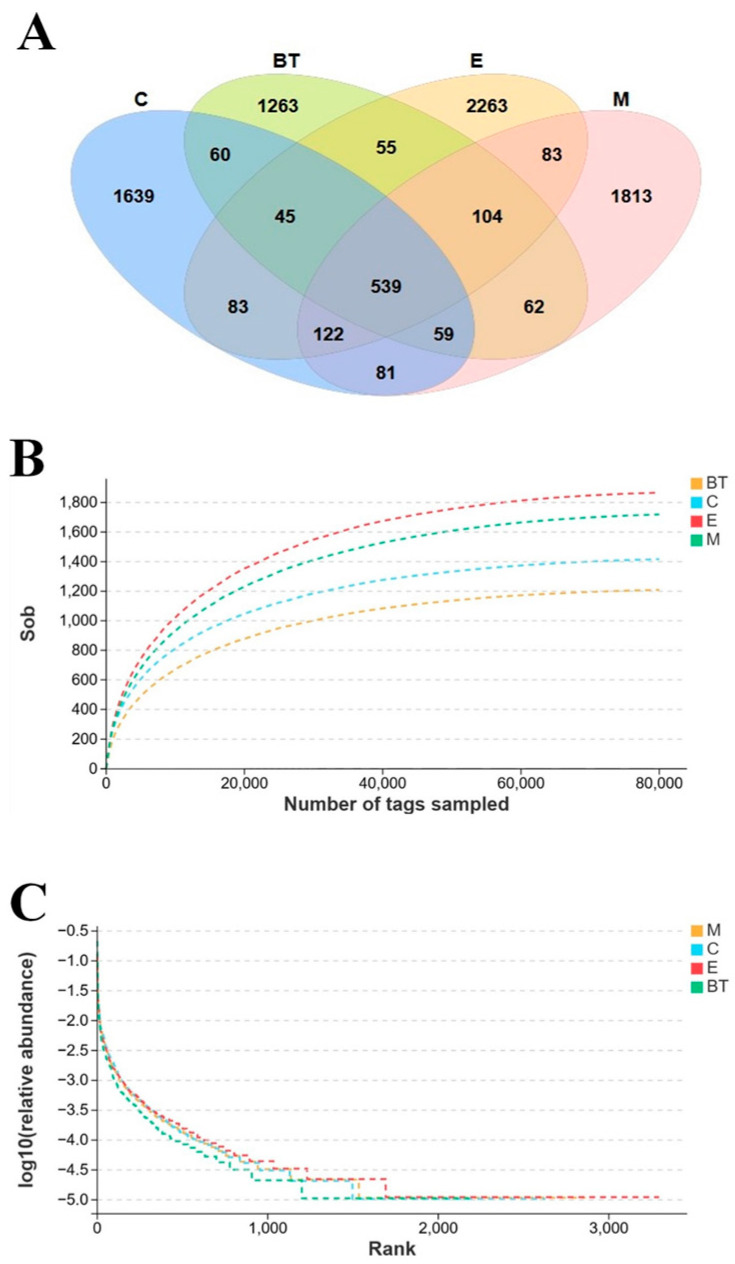
Sequencing data analysis and OTUs distribution. (**A**) Venn diagram showing the distribution of OTUs or sequence tags across the four groups. Numbers inside the overlapping and unique regions indicate shared and group-specific OTUs. Colors correspond to the experimental groups as follows: blue = Control (C), red = BT, blue-green/teal = E, and pink = M. (**B**) Rarefaction curve and (**C**) rank abundance curve for each group.

Microbial composition across the four groups was assessed through high-throughput sequencing of the 16S rRNA gene’s V3–V4 regions. A Venn diagram (Figure 9A) illustrates the distribution of operational taxonomic units (OTUs), with a total of 8188 OTUs identified across all samples. Among these, 539 OTUs were shared by all groups, while 2647, 2224, 3182, and 2863 unique OTUs were detected in the C, BT, E, and M groups, respectively.

Rarefaction curves and species accumulation curves for all samples showed broad consistency, with each group containing 20,000–80,000 qualified sequences (Figure 9B). This indicates that the sequencing depth and amount of data were sufficient to capture the basic microbial diversity of the samples. The rank–abundance curve shows a wide range and a low slope (Figure 9C), indicating high species abundance and evenness within the microbial communities, which accounted for ~5.21% of all OTUs.

These sequencing data characteristics confirm the reliability and comprehensiveness of the microbial community profiling, providing a solid foundation for subsequent analyses of alpha diversity, beta diversity, and taxonomic composition.

### 3.11. Gut Microbial Diversity Analysis

**Figure 10:**
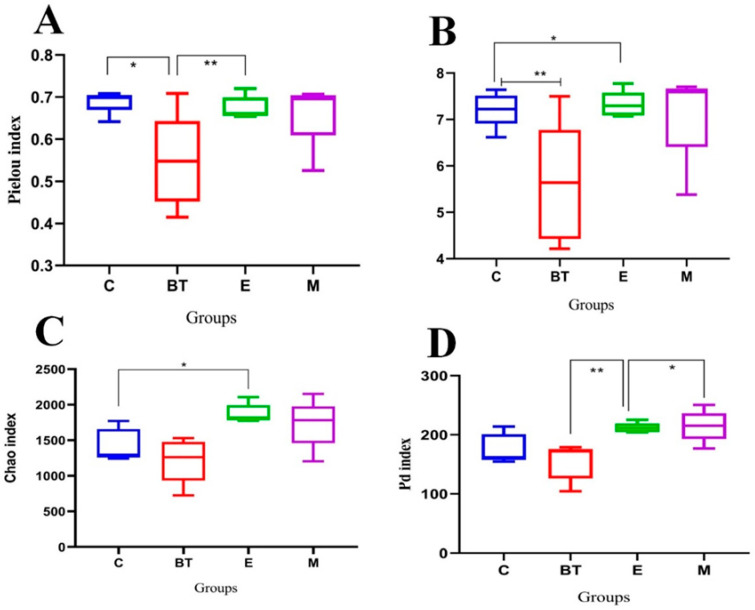
(**A**) Chao1, (**B**) Shannon, (**C**) Pielou, and (**D**) Faith_pd are the α-diversity indexes studied in this research. * *p* < 0.05 and ** *p* < 0.01.

Alpha diversity measures were applied to characterize variations in community richness, evenness, and phylogenetic structure of the gut microbiome in each experimental group (Figure 10A–D). The Faith’s PD index (a measure of phylogenetic diversity) was significantly lower in the *E. coli*-infected group (E group) compared to the *B. thetaiotaomicron* treatment group (*BT6* group) (Figure 10D). Additionally, the Chao1 (richness), Pielou (evenness), and Shannon (diversity) indices were higher in the control group (C group) than in the E group, with the Shannon and Pielou indices showing statistically significant differences (*p* < 0.05; Figure 10A–C). These results indicate that *E. coli* O157:H7 infection reduces gut microbial diversity and evenness, while supplementation with *B. thetaiotaomicron* enhances microbial community homogeneity and richness.

**Figure 11:**
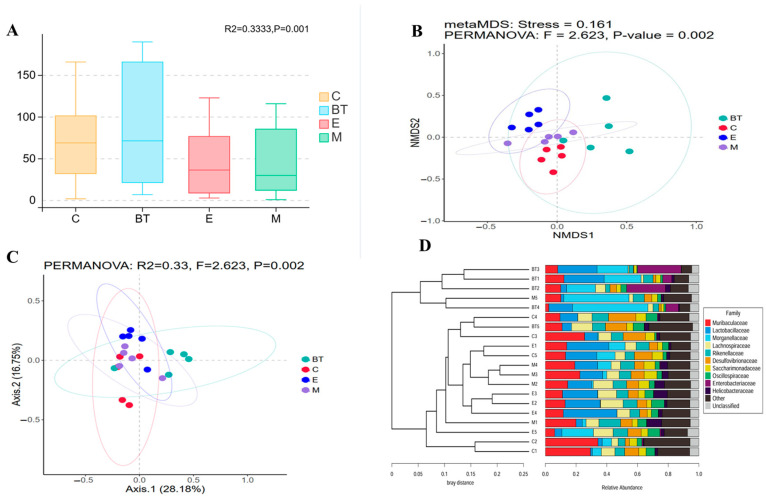
(**A**) PCoA plots based on Jaccard distances. (**B**). NMDS analysis. (**C**) The unweighted pair-group approach with arithmetic means. (**D**) PERMANOVA.

Beta diversity was assessed to examine structural differences in the microbial communities between groups. NMDS ordination, constructed using Bray-Curtis dissimilarities, showed clear separation in sample clustering among the different treatments, with a PERMANOVA test confirming significant overall differences (R^2^ = 0.33, F = 2.623, *p* = 0.002; Figure 11B). Principal Coordinates Analysis (PCoA) further demonstrated clear separation of microbial communities across groups, with the first axis explaining 28.18% of the total variation (Figure 11A). Pairwise PERMANOVA comparisons with Benjamini-Hochberg adjustment indicated that the beta diversity of fecal samples from the BT group was significantly different from that of the E group (adjusted *p* = 0.002; Figure 11C), reflecting substantial differences in microbial community structure between these two treatment groups.

Unweighted Pair Group Method with Arithmetic Mean (UPGMA) clustering dendrograms show that the similarity between samples increased as the branch length decreased (Figure 11D). Collectively, these beta diversity results confirm that *E. coli* O157:H7 infection disrupts the gut microbial community structure, while *B. thetaiotaomicron* (*BT6*) supplementation mitigates this dysbiosis and restores microbial community equilibrium.

### 3.12. Gut Microbial Composition Analysis

**Figure 12:**
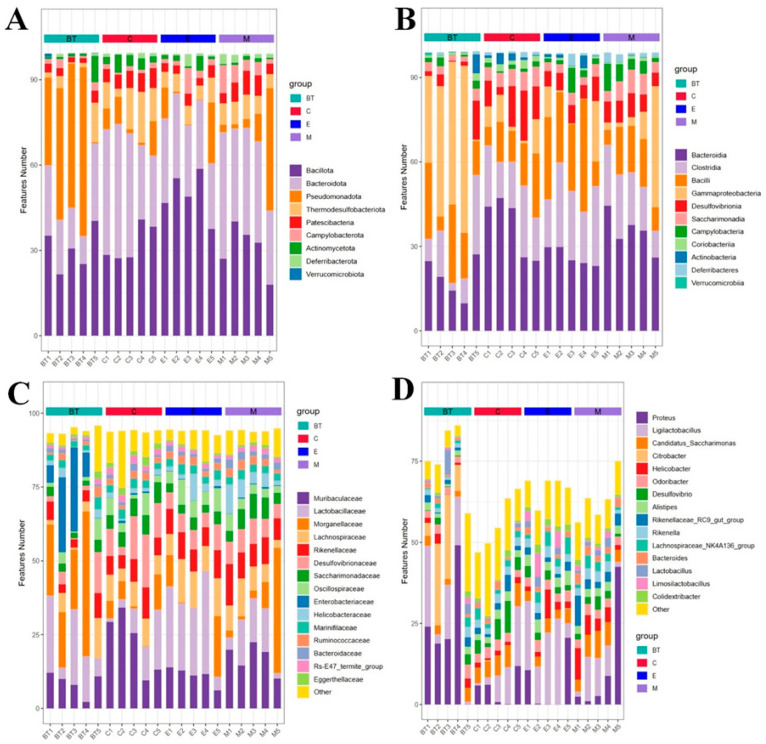
The structure of the gut flora across different taxonomic classifications. (**A**) Phylum, (**B**) class, (**C**) family, and (**D**) genus.

The gut microbial community composition differed significantly across the four experimental groups at the phylum, class, family, and genus levels (Figure 12A–D). At the phylum level, *Bacillota*, *Bacteroidota*, and *Pseudomonadota* were the dominant taxa in all groups, but their relative abundances varied markedly: in the control group (C group), they accounted for 32.51%, 37.21%, and 6.52%, respectively; in the *B. thetaiotaomicron* treatment group (BT group), 30.61%, 19.06%, and 37.53%, respectively; in the *E. coli*-infected group (E group), 49.45%, 26.36%, and 6.66%, respectively; and in the *B. thetaiotaomicron* + *E. coli* combination group (M group), 30.68%, 35.29%, and 11.98%, respectively.

At the class level, the C group was dominated by *Bacteroidia* (37.21%), *Clostridia* (18.37%), and *Bacilli* (13.93%). The E group showed high abundances of *Bacteroidia* (26.36%), *Bacilli* (25.31%), and *Clostridia* (23.66%), while the *BT6* group was characterized by *Bacteroidia* (35.29%), *Clostridia* (17.65%), and *Bacilli* (12.83%). Notably, the M group was distinctly characterized by *Gammaproteobacteria* (37.52%), *Bacteroidia* (19.06%), and *Bacilli* (17.64%) as the dominant classes.

At the family level, *Muribaculaceae* (22.37%), *Desul-fovibrionaceae* (11.17%), and *Lactobacillaceae* (8.81%) were enriched in the C group. The relative abundances of *Morganellaceae*, *Lactobacillaceae*, and *Enterobacteriaceae* were high in the BT group (22.45%, 15.44%, and 13.62%), whereas the group E was highly represented by *Morganellaceae*, *Lactobacillaceae*, and *Enterobacteriaceae* (22.51%, 11.16%, and 11.05%). The M group was characterized by *Muribacu-laceae* (17.26%), *Morganellaceae* (11.49%), and *Lactobacillaceae* (10.25%).

At the genus level, the C group was dominated by the *Ligilactobacillus* (17.14%), *Proteus* (6.32%), and *Alistipes* (3.51%). The *BT6* group had high abundances of Proteus (22.44%), *Citrobacter* (12.61%), and *Ligilactobacillus* (12.03%), while the E group was enriched with *Ligilactobacillus* (17.14%), *Proteus* (6.32%), and *Helicobacter* (3.26%). In the M group, the dominant genera were Proteus (11.48%), *Ligilactobacillus* (7.54%), and *Helicobacter* (5.15%).

**Figure 13:**
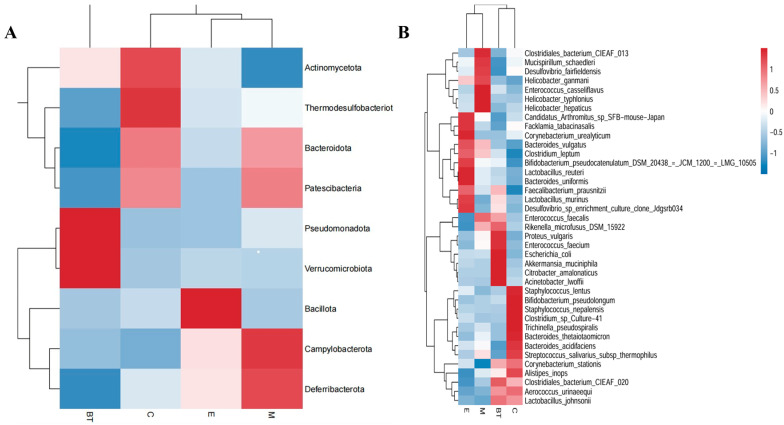
Hierarchically clustered heatmap of taxonomic analysis at the phylum level (**A**) and species level (**B**) for each group.

The relative abundances for phyla and species of bacteria were then examined in more detail using hierarchically clustered heatmaps, which highlight clear compositional patterns of bacterial communities corresponding to the four treatment groups (Figure 13A,B). These results collectively suggest that infection with *E. coli 0157:H7* disrupts the microbial composition of the gut, whereas supplementation with *B. thetaiotaomicron* modulates the microbial composition of the gut, increasing levels of beneficial taxa (*Lactobacillaceae* and *Muribaculaceae*) and decreasing levels of potentially harmful genera (*Citrobacter* and *Helicobacter*).

### 3.13. LEfse Analysis and T. Test Analysis

**Figure 14:**
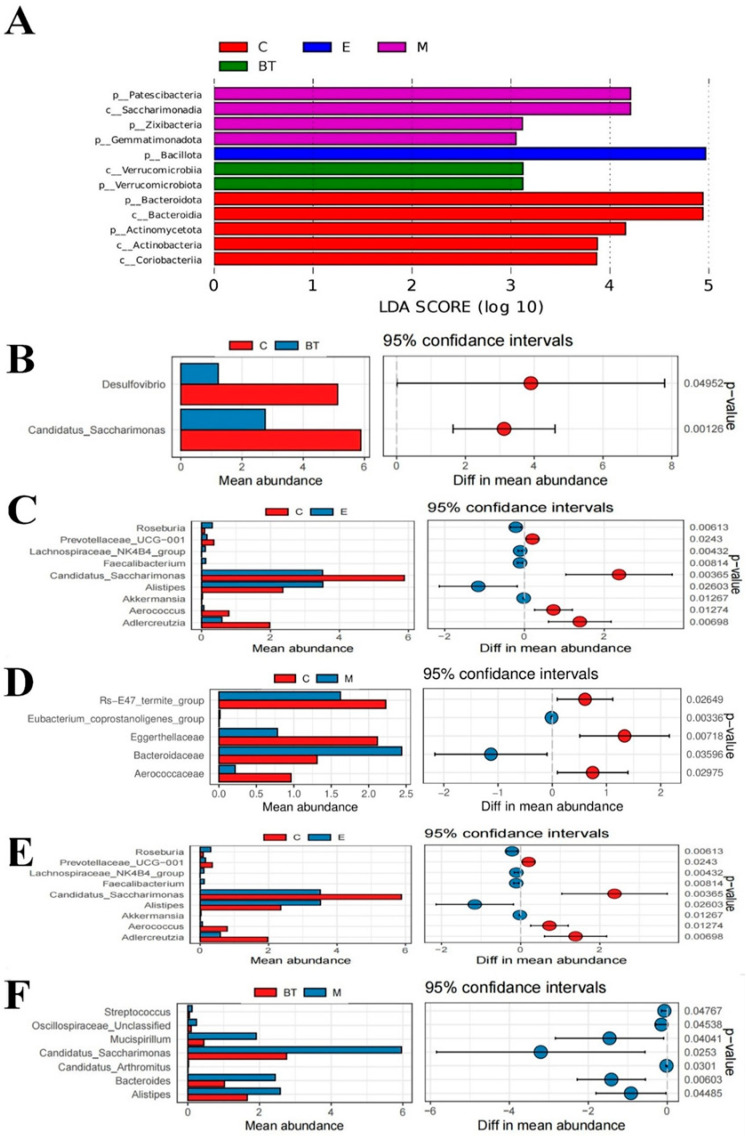
A list of abundant bacterial taxa is shown. (**A**) The linear discriminant analysis effect size (LEfSe) method was employed to identify bacterial taxa showing significant variation in relative abundance. Genus-level *T*-test analyses were conducted for the following comparisons: (**B**) C vs. BT group. (**C**) C vs. E group, (**D**) C vs. M group (**D**), (**E**) BT vs. E group, and (**F**) BT vs. M group.

LEfSe analysis was used to pinpoint bacterial taxa whose abundance differed significantly between the treatment groups (Figure 14A). In the *B. thetaiotaomicron* treatment group (BT group), *Verrucomicrobiota* (and its class *Verrucomicrobiia)* was the most prominently enriched taxon, with a linear discriminant analysis (LDA) score of 3.22. The control group (C group) showed significant enrichment of Bacteroidota (class *Bacteroidia*, LDA = 5.0), *Actinomycetota* (class Actinobacteria, LDA = 4.5), and *Coriobacteriia* (LDA = 3.87). The *B. thetaiotaomicron* + *E. coli* combination group (M group) had higher abundances of *Patescibacteria* (LDA = 4.35), *Saccharimonadia* (LDA = 4.35), *Zixibacteria* (LDA = 3.22), and *Gammaproteobacteria* (LDA = 3.20). In the *E. coli*-infected group (E group), *Bacillota* was the most significantly enriched phylum, with an LDA score of 4.98.

Analysis of fecal microbiota at the genus level also identified treatment-specific shifts (Figure 14B–F). Relative to the control (C) group, the *E. coli*-infected (E) group showed a significant rise in *Alistipes* (*p* < 0.05). In contrast, the co-administered (M) group exhibited higher levels of *Bacteroidaceae* (*p* < 0.05). Both the E and M groups displayed marked reductions in *Akkermansia*, *Aerococcus*, and *Eubacterium coprostanoligenes* compared to controls (*p* < 0.05).

The *BT6* group demonstrated a different modulation pattern: a decrease in *Desulfovibrio* versus the C group (*p* < 0.05); lower *Alistipes* levels than the E and M groups (*p* < 0.05); reduced *Enterococcus* compared to the E group (*p* < 0.05); and significantly less *Streptococcus* relative to the M group (*p* < 0.05). Together, these alterations indicate that *B. thetaiotaomicron* supplementation promotes microbial rebalance during *E. coli* O157:H7 infection by selectively regulating pivotal taxa.

### 3.14. Correlation of Gut Microbiota Among Different Groups

**Figure 15:**
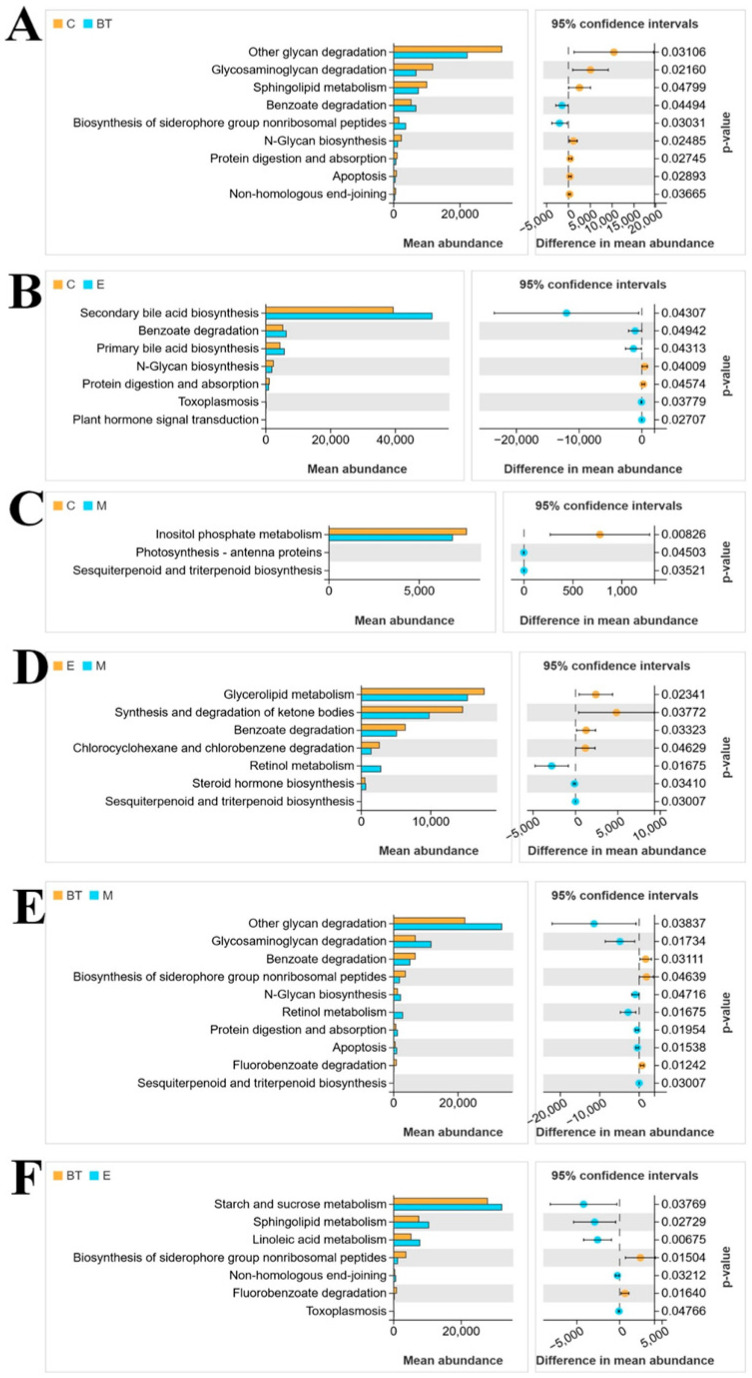
Analyzing the microbiota function between different groups, based on the KEGG pathway. (**A**) C vs. BT, (**B**) C vs. E, (**C**) C vs. M, (**D**) E vs. M, (**E**) BT vs. M, and (**F**) BT vs. E.

Functional attributes of the microbial communities were predicted using PICRUSt (Phylogenetic Investigation of Communities by Reconstruction of Unobserved States), which infers metagenomic functional potential from 16S rRNA gene sequences by mapping to KEGG pathway database. Six groups of KEGG pathways with significant differences (*p* < 0.05) in abundance among the experimental groups were identified (Figure 15). These pathways primarily reflected variations in substance metabolism, degradation, and synthesis processes across groups.

When comparing the control group (C group) with the *B. thetaiotaomicron* treatment group (BT group), nine pathways showed significant divergence (*p* < 0.05; Figure 15A). These included the degradation of glycans, glycosaminoglycans, sphingolipids, and benzoate, as well as the biosynthesis of N-glycans. For the C group versus the *E. coli*-infected group (E group), the secondary bile acid biosynthesis pathway, benzoate degradation pathway, and primary bile acid biosynthesis pathway were significantly upregulated (*p* < 0.05; Figure 15B). In the comparison between the C group and the *B. thetaiotaomicron* + *E. coli* combination group (M group), the inositol phosphate metabolism pathway exhibited a significant difference (*p* < 0.05; Figure 15C).

Seven KEGG pathways showed substantial changes between the E group and M group (Figure 15D). The M group displayed a significant increase in retinol metabolism (*p* < 0.05), while glycerolipid metabolism, synthesis, and degradation of ketone bodies, and benzoate degradation were not elevated. The comparison between the BT group and M group (Figure 15E) yielded similar results to the C group versus BT group comparison. When comparing the BT group with the E group (Figure 15F), starch and sucrose metabolism, sphingolipid metabolism, and linoleic acid metabolism were significantly increased (*p* < 0.05), whereas nonhomologous end joining was significantly decreased (*p* < 0.05).

## 4. Discussion

The identification and development of novel probiotic strains with targeted functions represents a significant focus in microbial research, driven by both industry demands and consumer interest. Within this field, *Bacteroides thetaiotaomicron* represents an important candidate, underlining the importance of isolating and characterizing strains of more natural origin with appropriate probiotic properties. The use of animal-derived probiotics offers unique benefits, combinations of which include the following: strengthening of host immunity, improving nutrition from feed, and supporting the management of infectious diseases.

Small ruminants such as goats are hardy and adaptable livestock. However, diarrheal illness in lambs and kids remains a significant cause of morbidity, mortality, and economic loss in goat production worldwide [22]. Enteropathogenic *E. coli* (EPEC) strains, including those belonging to the O157:H7 serotype, are recognized as important etiological agents of infectious diarrhea in young livestock, particularly neonatal and post-weaning lambs and kids. These pathogens adhere to intestinal epithelium, disrupt brush border morphology, and induce inflammatory diarrhea similar to the pathology observed in human infections [23].

To model this clinically relevant condition under controlled experimental conditions, we employed a well-established murine model using *E. coli* O157:H7 challenge in C57BL/6J mice, as previously described [23,29]. The mouse model offers several advantages for studying enteric infections: (1) it reproduces key features of bacterial enteritis including weight loss, diarrhea, intestinal barrier disruption, and inflammatory responses; (2) it enables controlled mechanistic investigation of host-pathogen interactions; and (3) it serves as a cost-effective and ethically acceptable platform for preclinical evaluation of potential probiotic interventions before progressing to target species trials [29]. This approach allowed us to rigorously evaluate the protective efficacy of caprine-derived *B. thetaiotaomicron* isolates under standardized conditions, providing a scientific foundation for future application in goat production systems.

Strain identification is usually a combination. Conventional approaches rely on phenotypic characteristics, such as colony and cellular morphology and biochemical profiles [39,40]. While informative, such methods tend to be labor-intensive and can involve subjective interpretation to limit their utility for definitive classification or high-throughput screening [41]. In contrast, alternative molecular methods such as 16S rDNA gene sequencing are fast and accurate for bacterial identification. This entails PCR amplification and sequencing of the 16S rDNA region, followed by comparison with public databases and phylogenetic analysis to determine taxonomic affiliation.

In our work, the initial examination of strains *BT6* and *BT7* showed that they were Gram-negative rods. Their phenotypic characteristics, evaluated with the use of standard manuals, were consistent with the genus *Bacteroides.* Sequencing of 16S rDNA was used to obtain definitive identification as *B. thetaiotaomicron.*

Growth kinetics is an essential property in the evaluation of potential probiotic strains. Rapid proliferation can contribute to a strain’s ability to establish itself within the gastrointestinal tract, potentially enhancing its capacity to compete with and exclude pathogens [42,43,44,45]. Our *B. thetaiotaomicron* isolates *BT6* and *BT7* exhibited logarithmic growth during the initial 0–12 h of culture, indicating promising proliferative capacity under in vitro conditions. This growth characteristic may support their ability to colonize the intestinal niche, although it is important to recognize that Bacteroides species possess fundamentally different physiological and metabolic strategies compared to Gram-positive lactic acid bacteria, including distinct cell wall architecture, fermentation pathways, and interactions with the host immune system.

Survival through the gastrointestinal tract is fundamental to the functioning of a probiotic. Tolerance to low gastric pH and intestinal bile salts is, therefore, an important benchmark [46]. On exposure of our strains to the simulated gastric juice (pH 2.5) and gastric juice (PG) (pH 2.5) for 3 h, our strains showed viability rates that ranged within 78.69–97.65%. Subsequent incubation at simulated intestinal fluid (pH 7.0) for 7 h yielded survival rates of 71.36–93.26%. Despite the harsh conditions, viable counts were maintained above 107 CFU/mL, demonstrating functional resilience. While other studies report variable survival at incredibly low pH (e.g., pH 2.0) [46,47], our strains showed a marked slowdown at pH 3.0 and no growth at pH 2.0. They were unaffected by 0.3% bile salts and grew well at pH 6.6, showing appropriate acid and bile tolerance for intestinal adaptation. The isolates were also tolerant of a range of temperatures (15–50 °C), where the survival rate was 35% at 15 °C and 57–60% at 37–50 °C, as previously reported for a potential probiotic bacterial strain [48].

Auto-aggregation is a desirable trait that has been associated with colonization of the intestine, as this behavior leads to aggregation and adhesion of bacteria to the epithelium [49] and can prevent the attachment of pathogens [50]. Another property correlated with adhesion potential is cell surface hydrophobicity. Prior studies report that the hydrophobicity values for certain *lactobacilli* and *bifidobacteria* range from around 39 to 58% [51,52,53,54]. In comparison, our strains *BT6* and *BT7* exhibited enhanced auto-aggregation and hydrophobic cellular behavior, indicating they are good candidates for gut colonization.

With growing restrictions on the use of antibiotics for animal livestock, probiotics are being explored as sustainable alternatives. No adverse effects were observed, and our strains exhibited no hemolytic activity. This strain exhibited a favorable safety profile in terms of characteristics. Furthermore, they showed direct antagonistic activity against *E. coli*, with an inhibition zone up to 22 mm. This result is consistent with other findings that various isolates of probiotic microbes can inhibit pathogens such as *Salmonella* and *Staphylocococus aureus* [55,56]. Antibiotic susceptibility profiling re-discovered that *BT6* and *BT7* were sensitive to most of the tested antibiotics, with intermediate sensitivity only to gentamicin and amikacin. This is in general agreement with other studies, although minor differences can be expected due to the specific strain or differences in methods [57].

Adhesion to intestinal mucosa is one of the determining factors for the persistence of probiotics and their interaction with the host [58,59]. Our in vitro assay proved that *BT6* and *BT7* adhered effectively to Caco-2 intestinal cells. This is consistent with studies that detected strong adherence of commercial *Lactobacillus* and *Bifidobacterium* strains [60,61], supporting their potential to colonize the gastrointestinal tract and possibly exclude pathogens [54]. In the in vivo model, histopathological examination, colon length, and the disease activity index (DAI) are standard parameters used to assess colitis [62,63].

Challenge with *E. coli O157:H7* successfully induced colitis, as demonstrated by the increase in DAI, a reduction in the length of the colon, and inflammation identified through histological examination. The observation of reduced water intake in the combination group (M) compared to controls, despite *E. coli* challenge, may initially appear paradoxical. However, this finding likely reflects the overall clinical improvement conferred by *B. thetaiotaomicron* (*BT6*) supplementation. Mice receiving the probiotic combination exhibited milder diarrhea (reflected in lower DAI scores), reduced intestinal inflammation, and improved general condition, which would diminish the thirst drive typically associated with fluid loss. Additionally, decreased water intake may indicate reduced disease severity and better maintenance of hydration status, consistent with the protective effects of *B. thetaiotaomicron* (*BT6*) on intestinal barrier function and fluid absorption. Similar observations have been reported in probiotic intervention studies where clinical improvement correlated with normalization of drinking behavior [29,30]. Treatment with *B. thetaiotaomicron* (*BT6*) reversed these changes; DAI was reduced, colon length was restored, and fewer inflammatory cells were able to infiltrate, suggesting a protective effect against colitis.

Cytokines play a central role in mediating immune and inflammatory processes [64,65]. The expression of cytokines is dysregulated in colitis, and levels are often related to the severity of the disease [66]. Cytokines that are pro-inflammatory, such as IL-6, IL-1β, and TNF-α, which are often produced by macrophages, are capable of increasing gut permeability and promoting inflammation [67,68]. It is well documented that their levels increase in *E. coli*-induced colitis [69]. In contrast, the anti-inflammatory cytokine IL-10 is immunosuppressive. Our results show that *B. thetaiotaomicron* (*BT6*) administration modulated this balance, decreasing pro-inflammatory IL-6, IL-1β, and TNF-α and increasing IL-10, suggesting a mechanism for its anticolitic effect.

Oxidative stress is another aspect of colitis pathology. Biomarkers such as MDA (lipid peroxidation product), antioxidant enzymes (SOD, CAT, GSH-Px), and energy metabolites (ATP) are useful indicators. Additionally, levels of reactive oxygen species (ROS) were measured as direct markers of oxidative stress [70,71]. *E. coli* O157:H7 infection induced marked oxidative imbalance, as evidenced by increased levels of MDA and ROS, elevated activities of the antioxidant enzymes SOD and CAT, and decreased levels of GSH (Figure 7). Interestingly, colonic ATP content was significantly elevated in infected mice compared to controls. While this may appear counterintuitive, increased ATP production has been reported during acute inflammatory responses and may reflect mitochondrial compensatory mechanisms in response to infection-induced cellular stress. Epithelial cells challenged with pathogens can upregulate oxidative phosphorylation to meet increased energy demands for barrier repair, immune signaling, and cellular survival. Alternatively, this elevation could result from reduced ATP utilization due to infection-induced inhibition of ATP-dependent processes, or from shifts in microbial contributions to the luminal ATP pool. Treatment with *B. thetaiotaomicron* (*BT6*) significantly reduced ATP levels toward control values, suggesting restoration of normal energy homeostasis. These findings underscore the complex relationship between energy metabolism and host defense during enteric infection. This is in agreement with the established role of probiotics in improving host antioxidant defenses [72], which is consistent with an anti-inflammatory profile [73].

Our study further showed that *B. thetaiotaomicron* (*BT6*) intervention improved overall gut microbiota diversity and restored gut composition changed by *E. coli* infection. Functional prediction analyses indicated that this intervention was also associated with the positive effect of stable metabolic pathways involved in nutrient utilization and environmental sensing. These findings suggest that beyond its direct anti-inflammatory and antioxidant effects, *B. thetaiotaomicron* plays a beneficial role in intestinal health by promoting a more balanced and functional microbial ecosystem.

Finally, we note several limitations, including: (1) We acknowledge that OD600 measurements reflect total bacterial cell mass and do not distinguish between live and dead cells. Therefore, these data are interpreted as an indicator of overall culture density rather than absolute viability. We now recommend that future mechanistic studies utilize CFU assays or flow cytometry-based live/dead staining to confirm bactericidal activity. (2) This study used a moderate number of mice, and validation in larger studies would provide potential results. (3) Only one pathogenic *E. coli* serotype (O157:H7) was tested; other clinically relevant strains should be used in future work (4). The assessment was focused on the short-term effects, and the long-term dynamics of colonization and care need further examination. (5) Future studies should also utilize a mechanistic approach, for example, utilizing gnotobiotic models or bacteria mutants, to specifically define the interactions between the host and microbes that lead to the observed benefits.

## 5. Conclusions

This study demonstrates that *Bacteroides thetaiotaomicron* strains *BT6* and *BT7*, isolated from healthy goats, possess potential probiotic properties, including excellent gastrointestinal tolerance, high cell surface hydrophobicity, and effective antagonistic activity against *E. coli* O157:H7. In a murine infection model, supplementation with *B. thetaiotaomicron* (*BT6*) significantly alleviated diarrhea, improved growth performance, and protected colon mucosal integrity. These benefits were mediated through modulation of inflammatory cytokines (downregulation of IL-1β, IL-6, TNF-α; upregulation of IL-10), enhancement of antioxidant capacity (reduced MDA, restored SOD, CAT, and GSH levels), and restoration of gut microbial homeostasis—characterized by increased beneficial taxa (*Ligilactobacillus*, Muribaculaceae) and suppression of harmful genera (*Helicobacter*, *Citrobacter*), alongside elevated propionic and butyric acid levels.

## Figures and Tables

**Table 1:** Survival of potential *B. thetaiotaomicron* probiotic isolates in artificial gastric and intestinal juices (mean ± SD).

Strains	Initial Count of Log cfu/mL (0 h)	SGF (Log CFU/mL) (3 h)	Survival Rate (%)	SIF (Log CFU/mL) (7 h)	Survival Rate (%)
*BT6*	0.28 ± 0.12	0.31 ± 0.07	78.69%	0.35 ± 0.35	71.36%
*BT7*	0.26 ± 0.05	0.37 ± 0.02	97.65%	0.41 ± 0.16	93.26

**Table 2:** Auto-aggregation and cell surface hydrophobicity abilities of potential probiotic *B. Thetaiotaomicron* isolated from healthy goat feces (mean ± SD).

Strain		Auto-Aggregation (%)		Hydrophobicity (%)	
Time				Xylene	Chloroform
	0 h	4 h	24 h	1 h	1 h
*BT6*	0.56 ± 0.41	1.13 ± 0.43	1.07 ± 0.46	67%	72%
*BT7*	0.40 ± 0.15	0.64 ± 0.33	0.91 ± 0.48	61%	93%

**Table 3:** Antibiotic susceptibility results of two selected strain isolates identified as best probiotics.

Strain	*BT6*	*BT7*	
Antibiotic	Zone Inhibition	Antibiotic	Zone Inhibition
Enrofloxacin	≤20	Enrofloxacin	≤22
Norfloxacin	≤19	Norfloxacin	≤21
Doxycycline	≤21	Doxycycline	≤23
Amoxicillin	≤20	Amoxicillin	≤20
Ciprofloxacin	≤19	Ciprofloxacin	≤21
Tetracycline	≤23	Tetracycline	≤25
Penicillin	≤25	Penicillin	≤25
Gentamicin	≥13	Gentamicin	≥11
Florfenicol	≤20	Florfenicol	≤19
Amikacin	≥12	Amikacin	≥10

## Data Availability

The original contributions presented in this study are included in the article/Supplementary Materials. Further inquiries can be directed to the corresponding author.

## Supplementary Materials

The following supporting information can be downloaded at: https://www.mdpi.com/article/10.3390/vetsci13040324/s1, Figure S1. Changes in pH value over time in cultures supplemented with different Bacillus siamensis isolates (BT1–BT15) compared to the untreated control during co-culture with *E. coli*. Figure S2. Agarose gel electrophoresis of PCR productsfrom samples. Lane M: DNA molecular weight marker (with the 1500 bp band indicated by arrow). Lanes 1–15: primer combinations. Expected product size: ~1500 bp. Gel: 1% agarose, stained with ethidium bromide/SYBR Safe, visualized under UV transillumination. Table S1. Biochemical characterization of tow strains. Table S2. Safety assessment of probiotic bacteria isolates.
