# Supplementary material for: Bacteroides thetaiotaomicron (BT6) Restores Intestinal Homeostasis in Escherichia coli O157:H7-Challenged Mice
Source: Vet Sci. 2026 Mar 27;13(4):324. doi: 10.3390/vetsci13040324 (PMC13120422; doi:10.3390/vetsci13040324)
Supplement: Supplementary file 1 [file vetsci-13-00324-s001.zip › Tables S1 and S2.pdf]

TableS1 : Biochemical characterization of tow strains

BT6		BT7	
Cellobiose	+	Cellobiose	+
Maltose	++	Maltose	+
Salicin	+	Salicin	++
Sucrose	+	Sucrose	++
Raffinose	+	Raffinose	+
Lactose	+	Lactose	+
Glucose	++	Glucose	+
Sorbitol	+	Sorbitol	-
Inulin	-	Inulin	-
Catalase	+	Catalase	+
Methyl red	+	Methyl red	+
Indole	+	Indole	+
Voges -proskauer	-	Voges -proskauer	-
citrate	-	Citrate	-

TableS2 :- Safety assessment of probiotic bacteria isolates

**Strains**	**Hemolytic activity test**
**BT1**	ß
**BT2**	α
**BT3**	α
**BT4**	ß
**BT5**	α
**BT6**	γ
**BT7**	γ
**BT8**	ß
**BT9**	ß
**BT10**	ß
**BT11**	ß
**BT12**	α
**BT13**	ß
**BT14**	ß
**BT15**	ß
