# Supplementary figures and images for: Bacteroides thetaiotaomicron (BT6) Restores Intestinal Homeostasis in Escherichia coli O157:H7-Challenged Mice
Source: Vet Sci. 2026 Mar 27;13(4):324. doi: 10.3390/vetsci13040324 (PMC13120422; doi:10.3390/vetsci13040324)

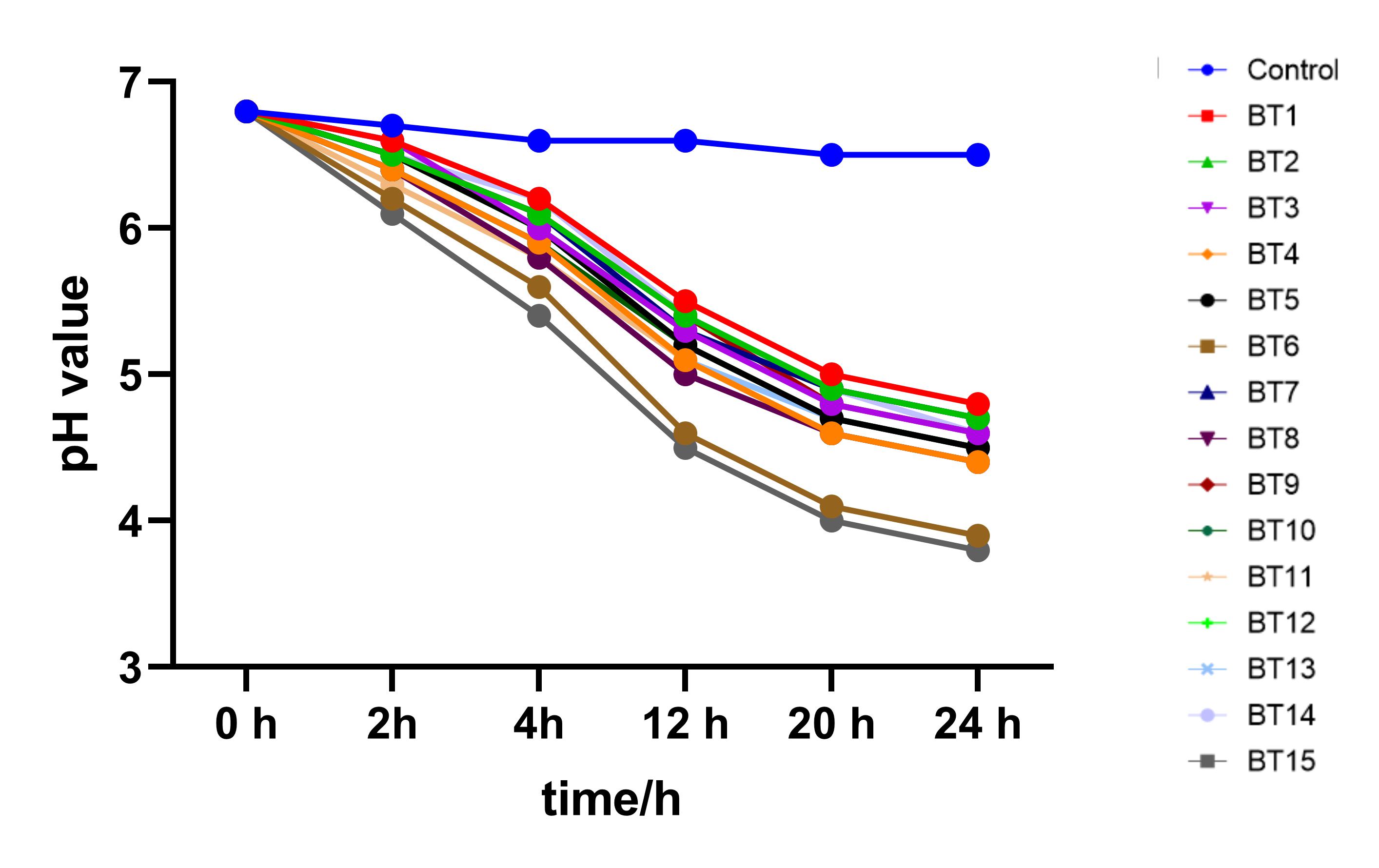

Supplement: Supplementary file 1 [file vetsci-13-00324-s001.zip › Figure S1.jpg]

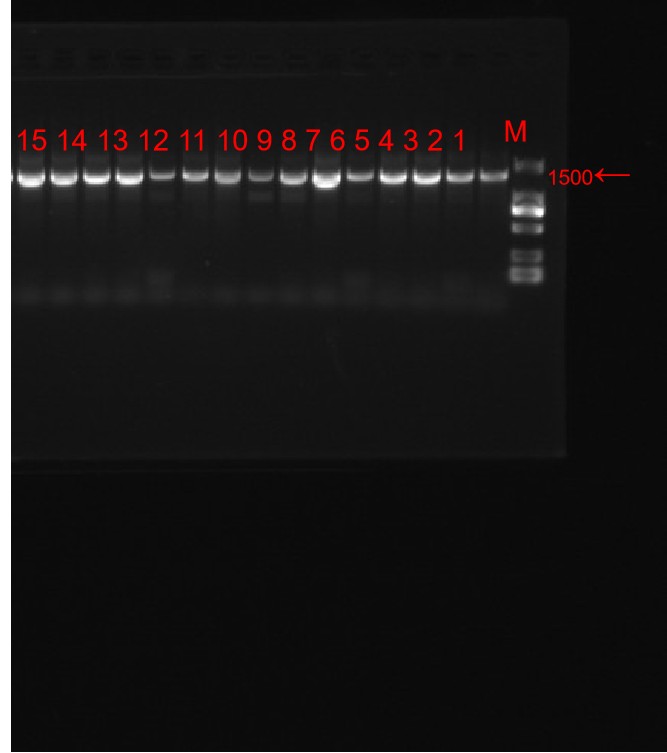

Supplement: Supplementary file 1 [file vetsci-13-00324-s001.zip › Figure S2.jpg]
